# Building and Raising Land: Mud and Vegetation Effects in Infilling Estuaries

**DOI:** 10.1029/2021JF006298

**Published:** 2022-01-17

**Authors:** S. A. H. Weisscher, K. Van den Hoven, H. J. Pierik, M. G. Kleinhans

**Affiliations:** ^1^ Faculty of Geosciences Utrecht University Utrecht The Netherlands; ^2^ Now at Water Systems and Global Change Group Wageningen University Wageningen The Netherlands; ^3^ Now at Ministry of Education, Culture and Science Cultural Heritage Agency Amersfoort The Netherlands

**Keywords:** estuaries, flume experiments, mudflat, salt marsh, steady state

## Abstract

Many Holocene estuaries were infilled to form convergent, single‐channel systems, while others remained partially or wholly unfilled. This difference in the degree of infilling depends partly on the balance between fluvial and coastal sediment input and the hydrodynamics that can export sediment. However, it remains unclear to what degree this balance is tipped by mud supply and eco‐engineering vegetation, and by what planform patterns the infilling proceeds. This study aims to explore experimentally how mud and vegetation change the degree and process of infilling, elevate and merge bars above intertidal levels and affect the planform of estuaries. To this end, three experiments were conducted in the Metronome, a flume that tilts periodically to create tidal currents, wherein forced tidal asymmetry resulted in net importing estuaries. In the second and third experiments, mud was supplied and in the third experiment seedlings were released of three vegetation species with eco‐engineering traits at a laboratory scale. With only sand, the estuary fills sufficiently to form a multi‐channel pattern with intertidal bars. Both mud and vegetation settle on intertidal bars and on the fluvial bay‐head delta, thereby contributing to bar stabilization and further estuary infilling, pointing at effective strategies to keep up with future sea‐level rise. This reduces channel mobility and effectively narrows the summed subtidal channel width toward an ideally converging funnel shape. This seems especially effective where vegetation stabilizes the mud. The experiments suggest that a range of steady states exists between the end‐members of an unfilled and a completely infilled, ideal estuary.

## Key Points

- Subtidal channels develop toward a convergent planform regardless of tidal bar and tidal flat geometry
- Mud and vegetation affect estuary development by filling intertidal area and confining channels
- A continuum of steady states exists between the classic unfilled estuary and the ideal, convergent estuary

## 1 Introduction

**Figure 1:**
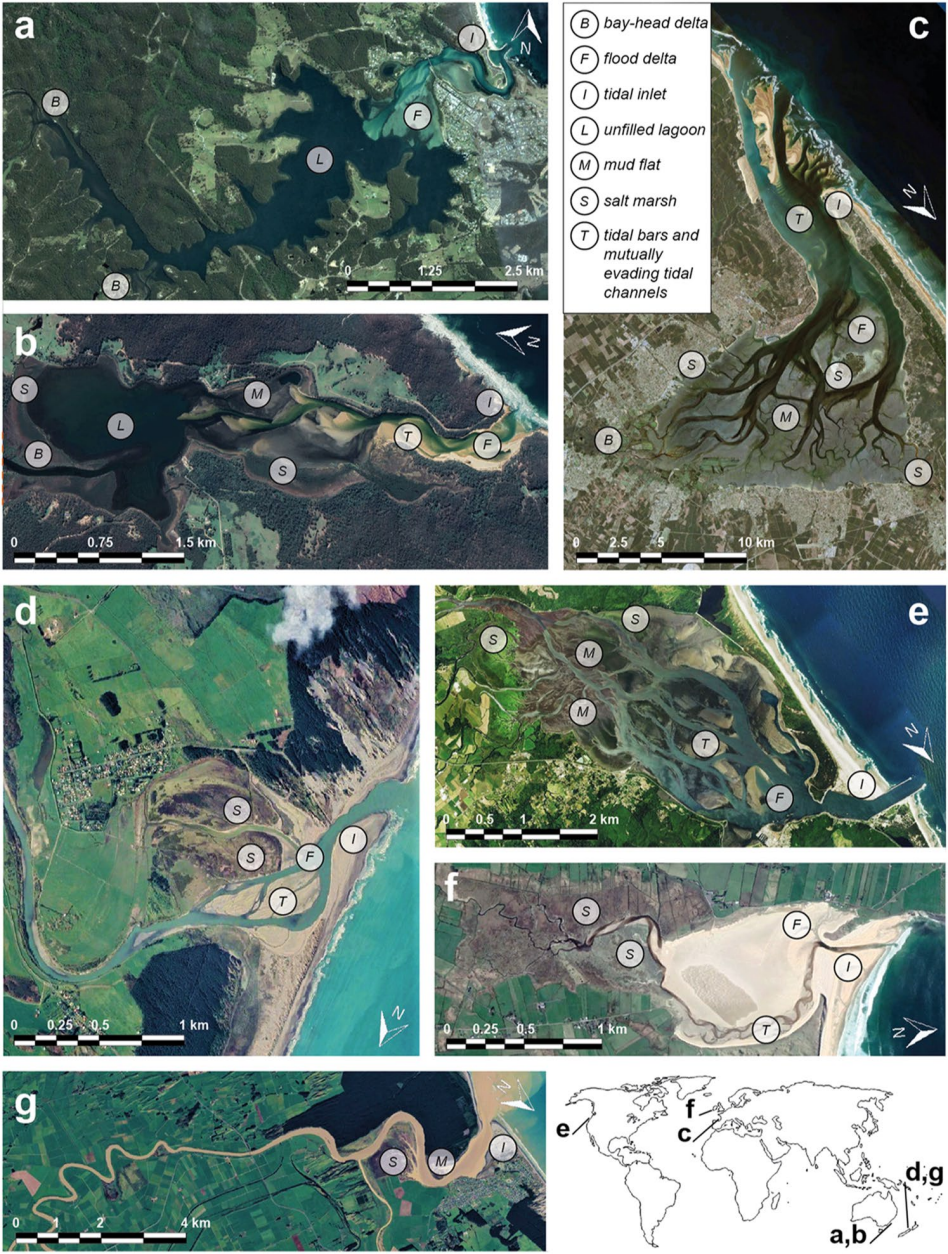
Estuaries ranging from unfilled to infilled on different spatial scales. (a) Wagonga Lagoon (AUS) is an unfilled estuary (ria coast) with a large flood‐tidal delta and two small bay‐head deltas (on the left and bottom left). (b) Wapengo Lagoon (AUS) is a largely unfilled estuary (ria coast) with tidal flats adjacent to the flood‐tidal delta (Nichol, 1991). (c) Arcachon Bay Estuary (FR) is a partly infilled estuary dominated by mudflats and has a small bay‐head delta. (d) Rangitikei River (NZ) and (g) Manawatu River (NZ) are both infilled estuaries (Clement et al., 2017). (e) Tillamook Bay Estuary (USA) is a partly infilled estuary with bars/flats and has a small bay‐head delta. (f) Carromore Lacken (IR) is a largely infilled estuary bounded by salt marshes and peatlands. In all images, river inflow is from the left and the sea is on the right.

As sea‐level rise decelerated during the Middle Holocene, many estuaries started to infill and formed new land (e.g., Clement et al., 2017; De Haas et al., 2018, 2019; Nichol, 1991; Roy et al., 1980; Umitsu et al., 2001; Van der Spek, 1995; Vos, 2015). Yet, other estuaries were only partly infilled or remained unfilled, in part due to a lack of sediment (Figure 1; e.g., Allen, 2000; Roy et al., 1980; Zecchin et al., 2009). These observations led to the recognition of two contrasting kinds of steady‐state estuaries. The first steady state is an unfilled estuary with a lagoonal basin that is largely below the threshold of motion (Roy et al., 1980). Here, the building of intertidal flats is inhibited by weak tidal currents, limited sediment availability and local, wind‐generated waves (e.g., Dalrymple et al., 1992). Unfilled estuaries generally form in deep (relative to tidal amplitude), drowned valleys with negligible fluvial and marine sediment input (e.g., the Wagonga and Wapengo Lagoons, AUS; Figures 1a and 1b; Roy et al., 1980). The second steady state is a completely filled estuary that ideally has a converging planform (e.g., the Manawatu River, NZ; Figure 1g; Dalrymple et al., 1992; Nichol, 1991; Roy et al., 1980). In an ideal estuary, landward convergence balances the decay of tidal energy by friction, resulting in a constant along‐channel tidal amplitude, channel depth and flow velocity (Savenije, 2015). Additionally, such an estuary has a balanced ebb‐ and flood‐directed sediment transport (Dronkers, 2017). A completely filled estuary with a convergent planform is considered a steady state (Dronkers, 2017; Savenije, 2015): a partly filled system develops flood‐dominant transport which promotes filling if sediment is supplied from the upstream river or the sea, whereas an over‐filled system develops ebb‐dominance and sediment export that may lead to delta formation (Dalrymple et al., 1994; Dronkers, 2017; Savenije, 2015).

### 1.1 Terminology of System Dynamics

Here, a steady state equilibrium is considered a geomorphic open system that self‐maintains a constant form with continuous throughput of material and energy despite all but the largest perturbations (Huggett, 2016; Thorn & Welford, 1994). In geomorphological nomenclature, this state is commonly also referred to as “dynamic equilibrium” (Zhou et al., 2017). However, general systems terminology prefers the term “steady state” for complex, open, entropy‐driven systems to “dynamic equilibrium,” because the latter is considered more appropriate for closed systems (Huggett, 2016; Thorn & Welford, 1994). Here, perturbations on the boundary conditions often merely move an estuary marginally away from its current steady state, to which it is “attracted.” Examples include both cyclic/seasonal perturbations such as the nodal cycle (Wang & Townend, 2012), and events such as river floods and storms (Swales et al., 2019; Townend et al., 2007).

Currently, the apparent dichotomy of the unfilled estuary and the ideal estuary raises the question whether estuaries have only two alternative steady states (or two “attractors”). On the geological timescale of sea‐level fluctuations, estuaries are considered ephemeral systems that will inevitably infill over time towards the presumed steady state of a single convergent channel, regardless of the transient patterns of lagoons, multi‐channel networks, mudflats and salt marshes (e.g., Dalrymple & Choi, 2007; De Haas et al., 2018; Lanzoni & Seminara, 2002). Then, the duration of infill depends on the amount of fluvial and coastal sediment input, which are functions of offshore and hinterland conditions. This reasoning, which assumes only two alternative steady states, would indicate that many present‐day systems (as in Figures 1c–1f) are not (yet) in a steady state, because they currently have multi‐channel planforms and intermediate degrees of infilling. On the centennial timescale, however, this reasoning seems contrary to observational evidence for some estuaries that showed no apparent development towards a convergent channel over the past centuries, despite the availability of sediment. For instance, the tidal channels in the Arcachon Bay Estuary (FR; Figure 1c) have remained static and equal in number for centuries despite the abundance of marine sediment (Allard et al., 2009; Fenies & Faugères, 1998). Another example is the Ems‐Dollard Estuary (NL/D), which has a high suspended sediment concentration but shows no clear signs of filling of tidal basins bordering the channel since the 1900s (e.g., Van Maren et al., 2016). Some classifications of estuaries and embayments also recognize various states between entirely unfilled and completely filled (Allen, 2000; De Haas et al., 2018), but these concepts raise the question whether these are attractor states or merely one of many possible transient states.

Certain numerical and physical models suggest that partly filled estuaries can be in steady state. Recent numerical modeling (Braat et al., 2017) and landscape‐scale experiments (Braat, Leuven, et al., 2019; Leuven, Braat, et al., 2018) showed the development of a steady state in sand‐bar dominated estuaries with multiple channels. These estuaries exhibited alternating wider sections with large bars and narrower sections with deep confluences, while the summed subtidal channel width developed towards a converging funnel shape (Leuven, Braat, et al., 2018). This was also observed in natural, multi‐channel, ingressive estuaries like the Western Scheldt (NL; Leuven, Braat, et al., 2018), as well as in infilling palaeo‐estuaries like the Old Rhine Estuary (NL; De Haas et al., 2018, 2019; Pierik et al., 2017) and the Shoalhaven River (AUS; Roy et al., 1980). Conceptually, the approach of isolating the subtidal channels reduces an estuary to a one‐dimensional system (Friedrichs & Aubrey, 1988; Speer & Aubrey, 1985), where the subtidal channels convey the flow momentum and the intertidal bars are conceptualized as having no other effect than being regions for water storage, just like shore‐connected bars and tidal flats (Leuven, Verhoeve, et al., 2018). Altogether, these examples in numerical modeling, experiments, and field observations point at the possibility of a continuum of steady states with different degrees of infilling.

### 1.2 Floodplain‐Forming Mechanisms

Mud and vegetation influence the local morphodynamics of estuaries through filling accommodation space and confining flow. More specifically, mud mainly settles as cohesive top layers on high‐intertidal bars and flanking flats, which confine and funnel ingressive estuaries and reduce the migration rate of channels and bars (Braat, Leuven, et al., 2019; Braat et al., 2017). As for salt marsh vegetation, this eco‐engineering agent establishes on high‐intertidal and supratidal areas, where the added hydraulic drag confines flow, stimulates sediment deposition and stabilizes the bed (e.g., Brückner et al., 2019; Lokhorst et al., 2018; Temmerman et al., 2007). These mechanisms through which mud and vegetation fill and confine estuaries are analogous to the floodplain‐forming mechanisms in rivers, where channel confinement causes a transition from braiding to meandering (e.g., Braudrick et al., 2009; Tal & Paola, 2010; Van Dijk et al., 2013; Weisscher et al., 2019). Accordingly, these mechanisms contribute to the building and raising of new land and are deemed necessary to reach the classic, ideally converging estuary (Savenije, 2015), whilst this converging planform is often imposed as non‐erodible banks in numerical studies (e.g., Dronkers, 2017; Lanzoni & Seminara, 2002; Olabarrieta et al., 2018; Savenije, 2015).

The filling of accommodation space (in geological terms) and flood storage space (in tidal terms) may have a second effect: it reduces the tidal prism and may increase flood‐dominance (e.g., Friedrichs, 1995; O’Brien, 1969). Previous studies hypothesized that quickly extending mudflats, salt marshes and peatlands may reduce tidal prism in a positive feedback loop (Beets & Van der Spek, 2000; De Haas et al., 2018). However, this hypothesis has not yet been clearly corroborated by the very few numerical studies and absent scale experiments that capture this long‐term morphodynamic process. Conversely, continued filling and in particular the expansion of intertidal flats may also induce a switch from flood‐to ebb‐dominance (Dronkers, 1986; Fortunato & Oliveira, 2005; Speer & Aubrey, 1985), which hampers further filling and changes the estuary from a sink to a source, as is expected, for example, for the Dee Estuary (UK; Moore et al., 2009). Accordingly, it remains unclear how mud and vegetation contribute to the two mechanisms of filling and confining wide lagoonal estuaries in drowned valleys on coastal plains and to what extent they modify the estuary planform during filling. For example, in North‐Western Europe, estuaries formed in the Middle Holocene on the drowned coastal plains by sedimentation and effects of vegetation, whilst sea‐level rise and human‐induced subsidence caused ingressions in the Late Holocene (De Haas et al., 2018; Gregoire et al., 2017; Pierik et al., 2017; Van der Spek, 1997).

To understand the morphological development of infilling estuaries, also in prospect of future sea‐level rise, there is an urgent need to determine whether the degree of filling and the resulting planform of estuaries develop towards one possible steady state or whether there can be a range of possible steady states. Furthermore, it is unclear whether and how mud and vegetation enhance the filling of estuaries and whether they are necessary to reach a converging estuary planform. Here, this study experimentally investigates the filling processes of alluvial estuaries with and without mud and vegetation and places the results in the context of recent studies on multi‐channel estuaries.

## 2 Methods

This study reports on three landscape scale experiments of entire estuaries. These experiments comprise a control with only sand, a second experiment with sand and mud, and a third with sand, mud and live vegetation but otherwise the same conditions. The present experiments of infilling estuaries differ from past experiments in three ways, detailed below. First, the initial bathymetry of the entire estuary is submerged during the tidal cycle except for the barriers, the banks and the uppermost area. This is different from Leuven, Braat, et al. (2018) and Braat, Leuven, et al. (2019), who started with narrow converging estuaries that expanded over time by exporting sediment, which represented Late Holocene ingressive estuaries (De Haas et al., 2018). The present experimental estuaries were based on pilot experiments in a smaller flume (see Text S2 in Supporting Information S1) and designed to have modest infilling over time by sand import so as to determine the added effects of mud and vegetation. To accomplish sand import, an overtide was added to the principal tide to create tidal asymmetry conducive to flood‐dominance (Kleinhans, Van Der Vegt, et al., 2017), thereby simulating conditions found, for example, in the North Sea (De Haas et al., 2018; Dronkers, 1986). Second, mud was added to two experiments on both the fluvial and tidal boundaries, following Braat, Leuven, et al. (2019). Third, seeds of three vegetation species were added to the flow in one experiment to act as eco‐engineering species (Lokhorst et al., 2019).

### 2.1 Experimental Setup and Procedure

Experiments were conducted in the tilting flume the Metronome. The 20 m long and 3 m wide flume tilts over the short central axis to generate tidal currents (Kleinhans, Van Der Vegt, et al., 2017). While a periodic sea water level fluctuation to create tides is at first sight closer to the situation in nature, as in previous studies (e.g., Reynolds, 1889; Stefanon et al., 2010), this classic method leads to a number of scale problems, namely: very low sediment mobility, ebb‐dominance and fast disappearing morphodynamics in the landward direction (Kleinhans, Van Rosmalen, et al., 2014). The novel tilting method solves these issues by tilting, which generates periodically alternating flows and mobile sediment by a slope in the landward direction during flood and a slope in the seaward direction during ebb (Kleinhans, Van Der Vegt, et al., 2017; Kleinhans, Van Rosmalen, et al., 2014; Kleinhans et al., 2015). Past experiments for the establishment of ingressive estuaries led, amongst others, to sand‐dominated estuaries with dynamic tidal bars and channels (Leuven, Braat, et al., 2018) and mudflats (Braat, Leuven, et al., 2019). Comparison between these experiments and natural estuaries showed that relative bar dimensions and the spatial mudflat distribution were largely similar.

**Figure 2:**
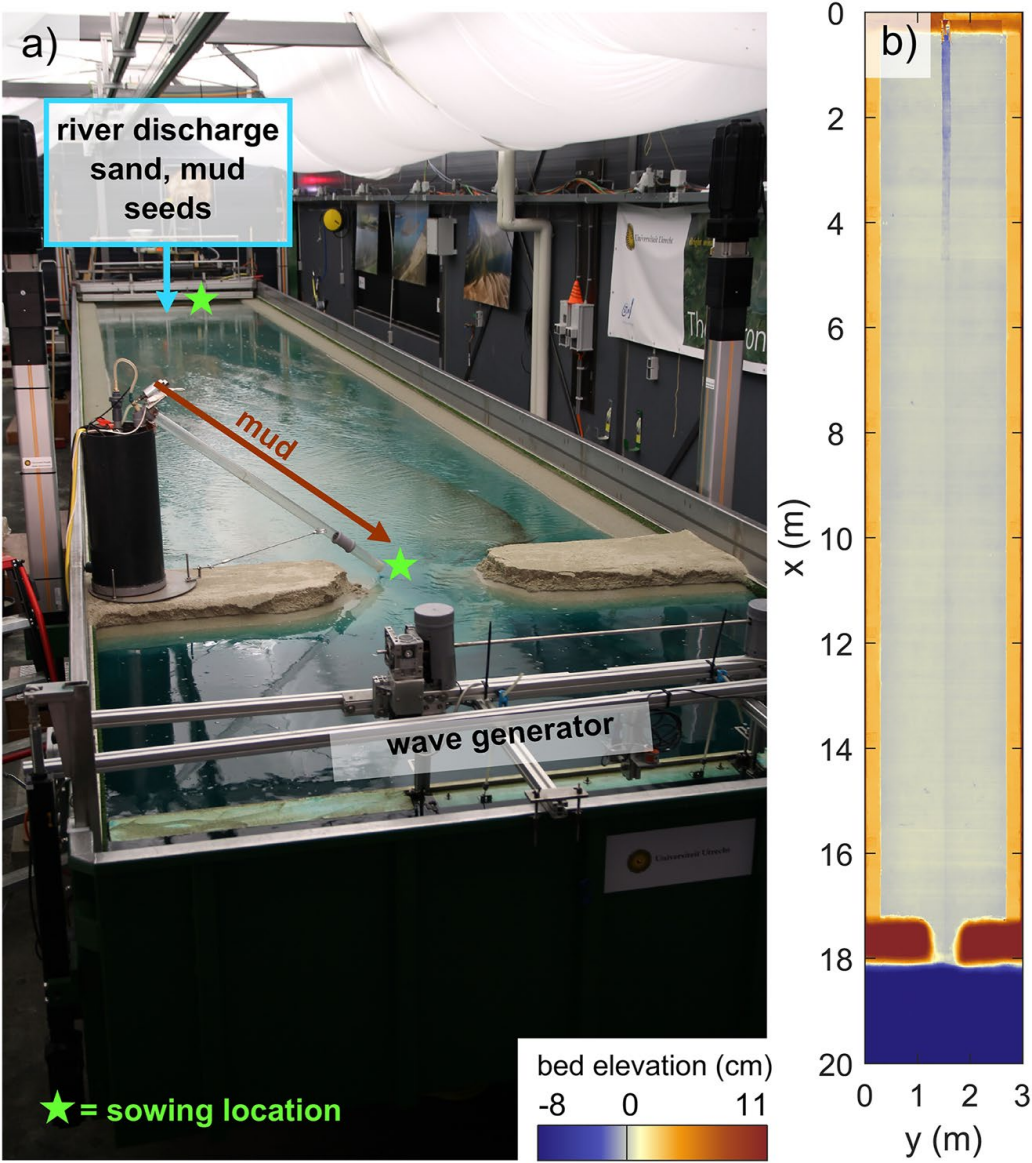
(a) The setup of the flume the Metronome. Crushed walnut was used as mud simulant and was released in pulses, one per tidal cycle, at the river and via a tube into the tidal inlet (brown arrow). Vegetation seeds were sowed via the river inflow and at the tidal inlet, after which they were distributed by tidal flow. (b) The initial bathymetry of the experiments was a rectangular estuary with a subtidal bed and high barrier islands. An initial river channel 0.16 cm wide and 1 cm deep was carved in the middle of the flat‐floored valley, decreasing in depth in the seaward direction.

The initial bathymetry was an idealized drowned river valley with a barrier coast (Figure 2). The initial bed consisted of sand with a median grain size *d*_50_ = 0.55 mm, a *d*_10_ = 0.32 mm and a *d*_90_ = 1.2 mm to prevent hydraulic smooth conditions and unrealistically large scours (Kleinhans, Leuven, et al., 2017). The sand bed was 17 m long, 3 m wide and had a bed thickness of 11 cm. Over the full length of the sand bed, a flat‐floored valley was carved of 2.4 m wide and 3 cm deep with a central, straight river channel of 16 cm wide and 1 cm deep in the upstream 5 m of the basin that gradually shallowed to 0 cm in the downstream direction. At the tidal inlet, two sandy barrier “islands” were constructed, each 1.15 m long, 0.6 m wide, and 19 cm thick with 45° slopes. These excessively large barrier islands acted as a source of marine sandy sediment, for it was infeasible to mimic alongshore drift to provide sufficient marine sediment input (Van Rijn, 1997). The entire bed was set at a typical fluvial valley slope in landscape experiments of 0.001 m/m (Kleinhans, Van Dijk, et al., 2014) by an offset tilt of the flume.

Flow conditions in the experiment were set to create a flood‐dominated estuary. In many estuaries, the combination of river inflow and a semidiurnal M2 tide leads to sediment export, while the addition of an M4 overtide causes net import of sediment (Dronkers, 1986). These boundary conditions for water flow were reproduced in the experiments by imposing tidal asymmetry on the tilting (Kleinhans, Van Der Vegt, et al., 2017). The principal tide was generated by tilting with a period of 40 s and a maximum slope of 0.006 m/m. The overtide had a period of 20 s and a maximum slope of 0.001 2 m/m (i.e., 20% of the principal tidal constituent) and had a phase difference of 90°. With the addition of the offset slope of 0.001 m/m, the tilting had a maximum slope of 0.006 2 m/m in landward direction during flood and 0.005 8 mm/m in seaward direction during ebb. There was free inflow and outflow of water at the seaward boundary, while a vertically moving weir ensured a constant water level at the shoreline of about 10 cm above the flume floor during tilting with a slight drop to 9 cm just after peak flood. The weir moved in the opposite phase of the tilt to keep the sea water surface horizontal while the flume bed tilted, to avoid emptying and filling of a tilting sea which would have caused large, unwanted water fluctuations at the shoreline (Kleinhans, Van Der Vegt, et al., 2017). Waves were generated by a horizontal paddle at the seaward boundary with a frequency of 2 Hz and an amplitude of about 0.3 cm during the flood phase. These waves contributed to the mobilization of sediment from the barrier islands and enhanced the landward transport on the ebb‐tidal delta (for scaling, see online supplement in Leuven, Braat, et al. (2018)). At the upstream boundary, a river discharge of 900 L/hr was added to the estuary during the ebb phase only.

Sand and mud were added to the river, both at a rate of 0.4 L/hr. The sand had the same grain size distribution as that of the initial bed, and the mud‐like material comprised three equal parts of the following grain size classes: 0.45–0.8, 0.8–1.3, and 1.3–1.7 mm. Crushed walnut shell with a density of 1,350 kg/m^3^ was used as mud simulant as in previous studies (Baumgardner, 2016; Braat, Leuven, et al., 2019). This lightweight material can be transported in suspension and is slightly cohesive after deposition. A second nutshell feeder supplied crushed walnut shells in the tidal inlet at the start of every flood phase at a rate of 0.4 L/hr to simulate the input of fines from the sea. As a result, a large range of sediment mobility was accomplished up to *θ* = 0.55 during peak flood, where the Shields number *θ* = *τ*/((*ρ*_*s*_ − *ρ*)*gd*_50_) with *τ* is bed shear stress, *ρ*_*s*_ and *ρ* are density of sand and water and *g* is the gravitational constant.

#### 2.1.1 Vegetation Protocol

Three species were selected with contrasting sensitivity to hydromorphological stresses and with contrasting eco‐engineering effects on flow and sediment transport in laboratory landscape experiments. Based on Lokhorst et al. (2019), who tested 19 vegetation species for growth rate, hydraulic drag and rooting strength in cut‐banks in Metronome‐like conditions, the following three species were chosen with different eco‐engineering traits: *Medicago sativa*, *Lotus pedunculatus*, and *Veronica beccabunga*. These species sprout quickly, remain fairly small in the absence of nutrients, and are sensitive to the miniaturized hydroperiod in the experiments. *Medicago*, commonly known as alfalfa, has seeds that are transported as bed load, typically establishes in the vicinity of channels, and reduces bank erosion by dense, branching rooting. Therefore, alfalfa resembles riparian vegetation on (tidal) levees (Lokhorst et al., 2019). *Lotus* and *Veronica* represent reed‐like and grass‐like marsh vegetation in North‐Western Europe, but their simple eco‐engineering traits are also similar to those in estuaries with mangroves (Lokhorst et al., 2019). Since the tiny *Veronica* seeds are transported in suspension, *Veronica* usually establishes in higher and more distal parts of the estuary.

In the experiment with vegetation, seeds were supplied after the initial 1,000 tidal cycles both via the river and the tidal inlet at a 500 tidal cycle interval until the end of the experiment. In the design, a coverage of half the estuary (i.e., 19 m^2^) was assumed to become vegetated by 6,000 tidal cycles with an average stem density of 10 stems/cm^2^ and a germination probability of 0.5. Per sowing event, 80,000 seeds of *Medicago*, *Lotus*, and *Veronica* each were supplied to the river and 80,000 seeds of *Lotus* and *Veronica* were supplied to the inlet.

Prior to sowing, seeds were soaked for 24 hr to stimulate sprouting. A sowing event commenced after dry bed scans (see Section 2.2) and 10 tidal cycles for initial wetting. Next, over the course of 25 cycles, the fluvial mixture of seeds was added to the river discharge at one spoonful per cycle. Subsequently, the marine mixture of seeds was introduced just landward of the tidal inlet in the main channel over the course of 10 cycles at one spoonful per cycle. Finally, tilting continued for another 25 cycles to spread the seeds, based on the design tidal excursion length and on earlier tests, resulting in a total of 70 tidal cycles per sowing event.

Following a sowing event, the flume was stopped at its offset slope of 0.001 m/m for 4 days to allow for sprouting. A river baseflow of 300 L/hr was applied to keep the tilted bed moist and the depth of the sea was increased to 10.8 cm to flood the channel and avoid sprouting in permanently submerged areas. Unfortunately, the high water table stimulated the growth of mold in low areas that received little river flow, increasing vegetation mortality (see Text S3 in Supporting Information S1 for the protocols to suppress algae and fungi). Therefore, after 3,500 cycles, the water depth of the sea was lowered to 10 cm during sprouting to minimize the mold.

### 2.2 Data Collection and Analysis

Time‐lapse photographs were shot by 7 AVG Mako (G‐419C) color cameras, positioned 3.7 m above the flume. The cameras had a resolution of 2,048 by 2,048 pixels and a fixed focal length of 12.5 mm, resulting in a pixel resolution on the sand bed of approximately 1.5 mm. Photographs were taken every tilting cycle at the end of flood when the flume was in a horizontal position. Before the images were stitched, the following corrections were applied to each image: noise removal, lens correction (vignette and distortion), geometric rectification and color corrections. Blue dye was added to the water to get an indication of water depth, which was extracted from the time‐lapse imagery to get an approximation of the bathymetry development throughout the experiments (see Weisscher et al., 2020, for review).

Digital elevation models (DEMs) were acquired by means of laser line scanning and stereo‐photography. The laser line scanner had a horizontal resolution of 0.75 mm and a vertical resolution of about 0.2 mm (Van Dijk et al., 2013). Since the accurate laser only covered the middle 2.4 m of the flume, somewhat less accurate stereo‐photography was used to acquire the remaining side parts of the flume. Prior to DEM acquisition, the flume was drained slowly to minimize disrupting the bed, and overhead photographs were taken of the dry bed for image classification. Subsequently, the bed was scanned and oblique photographs were taken with a digital single‐lens reflex camera and processed with structure‐from‐motion software (Agisoft PhotoScan). Increments between DEMs increased as the experiments progressed and morphological change reduced. First, DEMs were made with steps of 100 tilting cycles up to 500 cycles, followed by 8 DEMs with an interval of 250 cycles. Next, 5 DEMs were made with an interval of 500 cycles and the final 5 DEMs had an increment of 1,000 cycles, covering a total of 10,000 cycles. In the experiment with vegetation, DEMs were also taken just before a sowing event and right after a sprouting period to determine where vegetation had established or perished.

Flow measurements by instruments or particle image velocimetry is practically impossible in shallow flows, especially in the presence of vegetation. Therefore, flow data were acquired by the numerical model Nays2D, which was extended and validated by Weisscher et al. (2020) to simulate tidal flow in scale experiments (see Text S1 in Supporting Information S1). Nays2D takes as input a DEM and flow boundary conditions, applies the prescribed tilting motion of the flume and produces water depth and flow maps with a resolution of 2.5 by 2.5 cm over a tidal cycle. Vegetation was filtered out of the DEMs and added as increased hydraulic roughness, with a manning ranging from 0.02 s m^1/6^ for bare sand up to 0.167 5 s m^1/6^ for dense vegetation (i.e., 20 stems/cm^2^, 0.5 mm thick). The numerical output maps of Nays2D were made with increments of 1,000 cycles and were used to calculate sediment mobility, tidal prism, and residual currents. Furthermore, the estuary was classified into subtidal, intertidal, and supratidal depth ranges relevant for vegetation establishment and for basic interpretation of the hydrodynamics in terms of flood storage and momentum transfer (Friedrichs & Aubrey, 1988).

The tidal prism (*P*) and cross‐sectional area (Ω) show an empirical, near‐linear relation in natural tidal systems (e.g., Jarrett, 1976; Leuven, De Haas, et al., 2018; O’Brien, 1931, 1969), but scaled tidal experiments appear biased and do not fit on these relations (Mayor‐Mora, 1977; Seabergh et al., 2001; Stefanon et al., 2010). This bias is because the classic relationship Ω = *kP* ^*α*^, where *k* and *α* are constants (see Leuven, De Haas, et al., 2018, for review on parameter values), disregards friction effects that are much larger for small‐scale tidal systems (Kleinhans et al., 2015). The formulation by O’Brien (1969) accounts for these effects (also see the more complicated O’Brien‐Jarrett‐Marchi law: D’Alpaos et al., 2009; Stefanon et al., 2010). The O’Brien (1969) relation assumes that flood and ebb duration and peak flow velocities are equal and is given as follows:

(1) $$P=\int_{0}^{T}au\;dt$$

with

(2) $$u=U_{\max}\sin\left(\frac{2\pi t}{T}\right)$$

where *t* is time (s), *a* and *u* are the instantaneous cross‐sectional area (m^2^) and instantaneous, cross‐sectionally averaged flow velocity (m/s), *T* is the tidal period (s) and *U*_max_ is the maximum value of *u* (m/s). Solving the integral results in

(3) $$\Omega =cP^{\alpha }$$

where *c* = *π*/(*TU*_max_) and *α* = 1. As a result, Equation 3 allows for the comparison of natural tidal systems and experimental scale models (e.g., Seabergh et al., 2001).

## 3 Results

### 3.1 General Estuary Development

**Figure 3:**
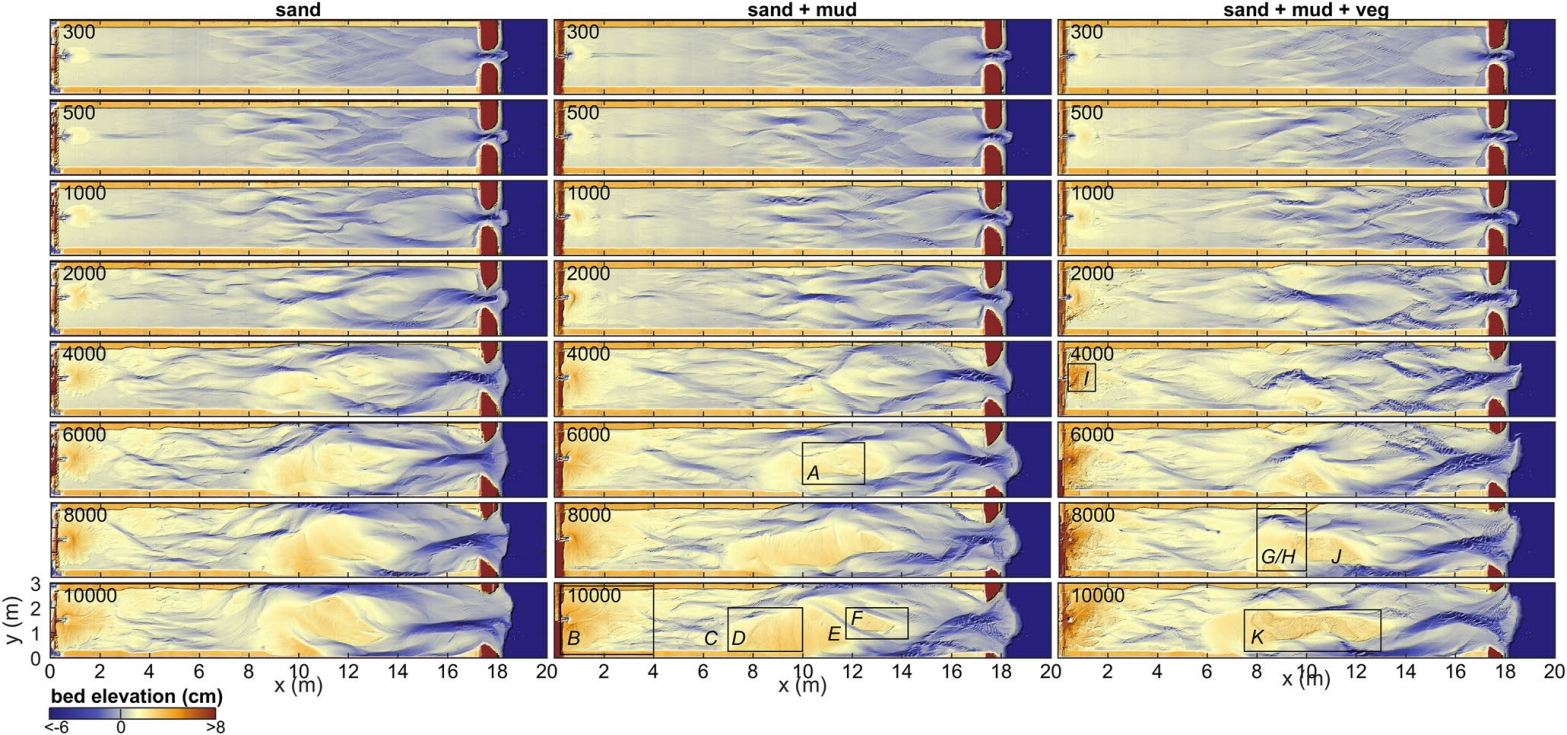
Morphological development of the infilling estuary experiments with only sand (left column), with mud (middle column), and with mud and vegetation (right column). Numbers indicate the tidal cycles. Letters correspond to the photographs in Figure 5.

The general development of the three experiments is described as follows. Initially, a low‐amplitude diamond‐shaped bar pattern emerged in the middle of the estuary. This pattern quickly transformed within the first 300–500 tidal cycles into short discontinuous tidal channels that ended in shoals on both ends (Figure 3). Meanwhile, a small ebb‐tidal delta and a large flood‐tidal delta formed near the inlet, and a bay‐head delta started forming near the landward boundary.

**Figure 4:**
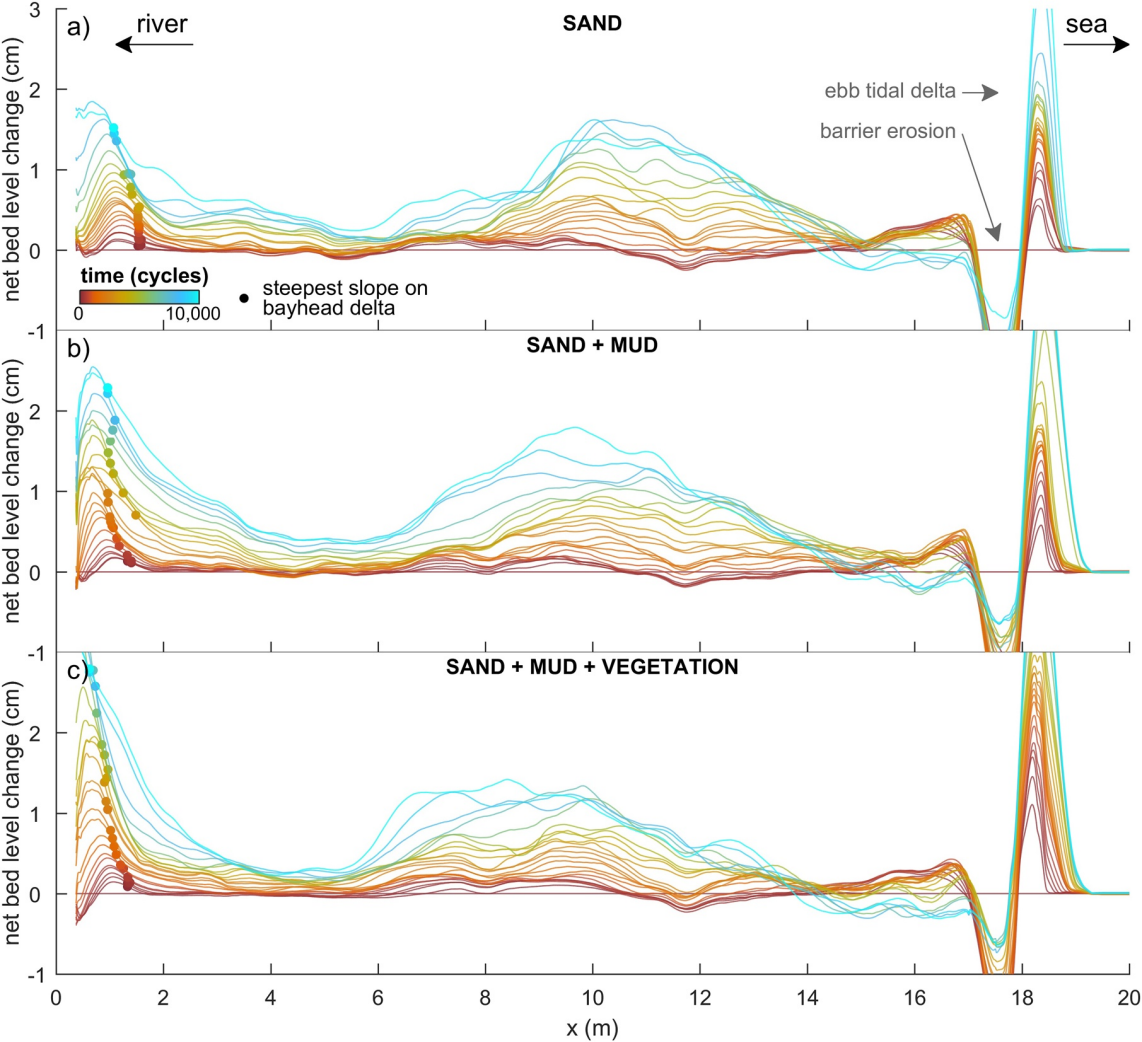
Mean cross‐sectionally averaged bed level along the estuary for the experiments with (a) only sand, (b) with mud, and (c) with mud and vegetation. Unreworked banks and the barriers were excluded. Dots represent the location of the steepest downward slope on the bay‐head delta (see text for discussion).

Over time, the channels in the estuary deepened, widened and extended in both the landward and seaward direction while bars merged. Landward extension of the tidal channels created a series of terminal lobes that increased the mean bed elevation in the upper half of the estuary (Figures 3 and 4), whilst the channels and shoals extending seawards were highly dynamic and coalesced with the flood‐tidal delta. Subtidal shoals typically separated ebb‐dominated and flood‐dominated channels, resulting in a mutually evasive tidal channel pattern. Continued growth and reorganization of the tidal channels led to the development of three main confluence‐diffluence nodes at *x* = 4, 10 and 17 m at around 2,000 tidal cycles. Between these confluences, there were on average two main channels flanking the banks of the estuary. It is at these locations that considerable bank erosion occurred, predominantly between *x* = 10 and 17 m where tidal currents were much stronger than between *x* = 4 and 10 m (Figure 3). At the shoreline, barrier erosion was mainly driven by flood currents and waves, and most eroded sediment was transported in the landward direction. The waves contributed to the development of a subtidal shield on the ebb‐tidal delta, flanked by flood‐dominant channels. The bay‐head delta aggraded and expanded by cyclic avulsion, starting with channel displacement, incision and extension after which the channel was backfilled and abandoned.

After about 6,000 tidal cycles, a large bar complex formed in the middle of the estuary by amalgamation of bars (Figure 3). This bar complex rose partly above the high water level and focused tidal flow into two main channels. Given that the bar complex became supratidal and the maximum water level around the bar dropped, this suggested that the collective flow friction reduced whilst the channels developed and deepened. As for the two main flanking channels, the one at *y* = 2.5 m (Figure 3) was more strongly ebb‐dominated than the other at *y* = 0.5 m in all three experiments. Over time, the growing bar complex was reworked repeatedly by cross‐cutting channels formed by water level differences between the main channels flanking the bar (Figure 3). This process caused sediment pulses into the main channels, and part of this sediment was transported in the landward direction, contributing to further infill (Figure 4).

### 3.2 Mud Distribution

**Figure 5:**
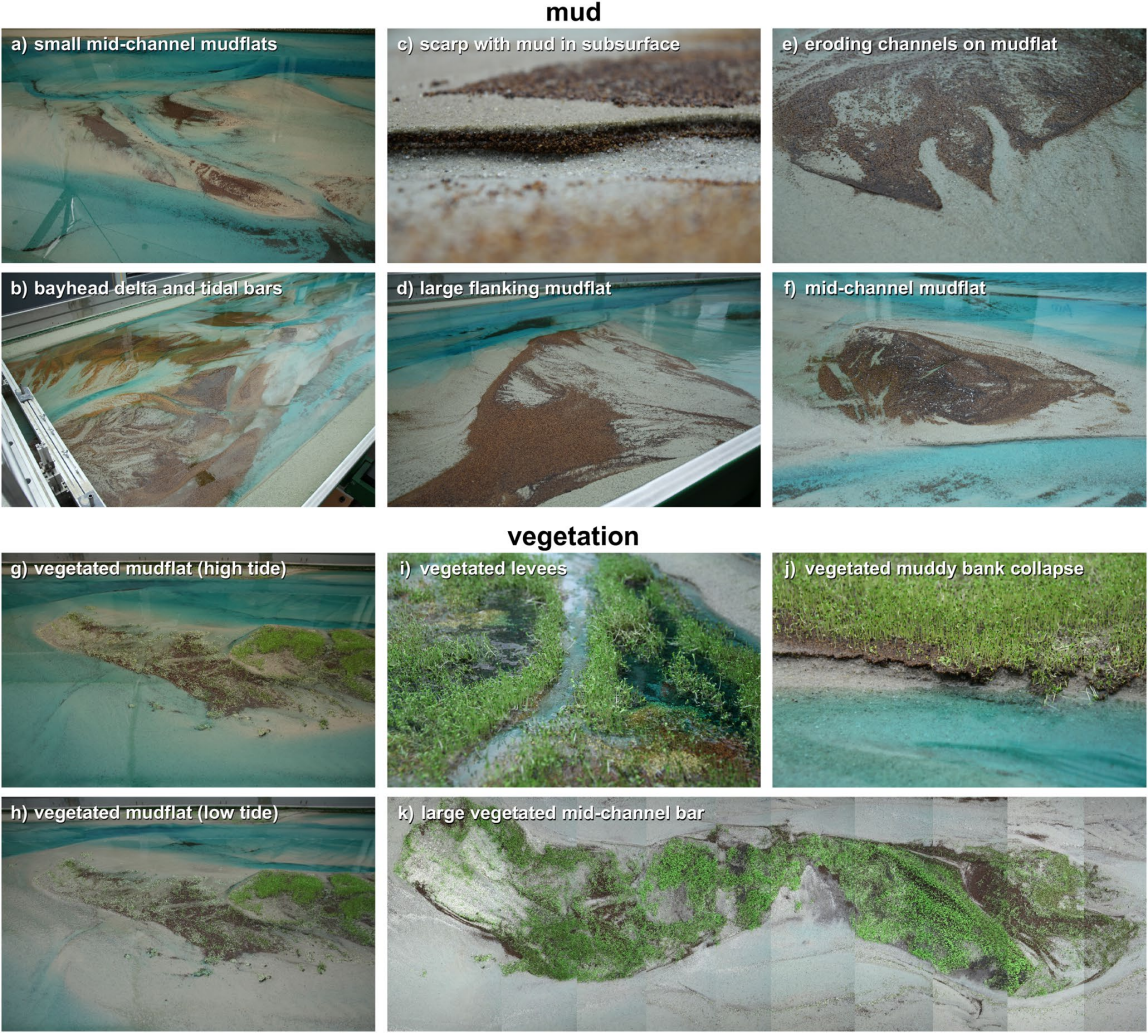
Photographs of (a–f) mud deposits and (g–k) vegetation in the experiments. The corresponding locations in the flume are indicated in Figure 3.

**Figure 6:**
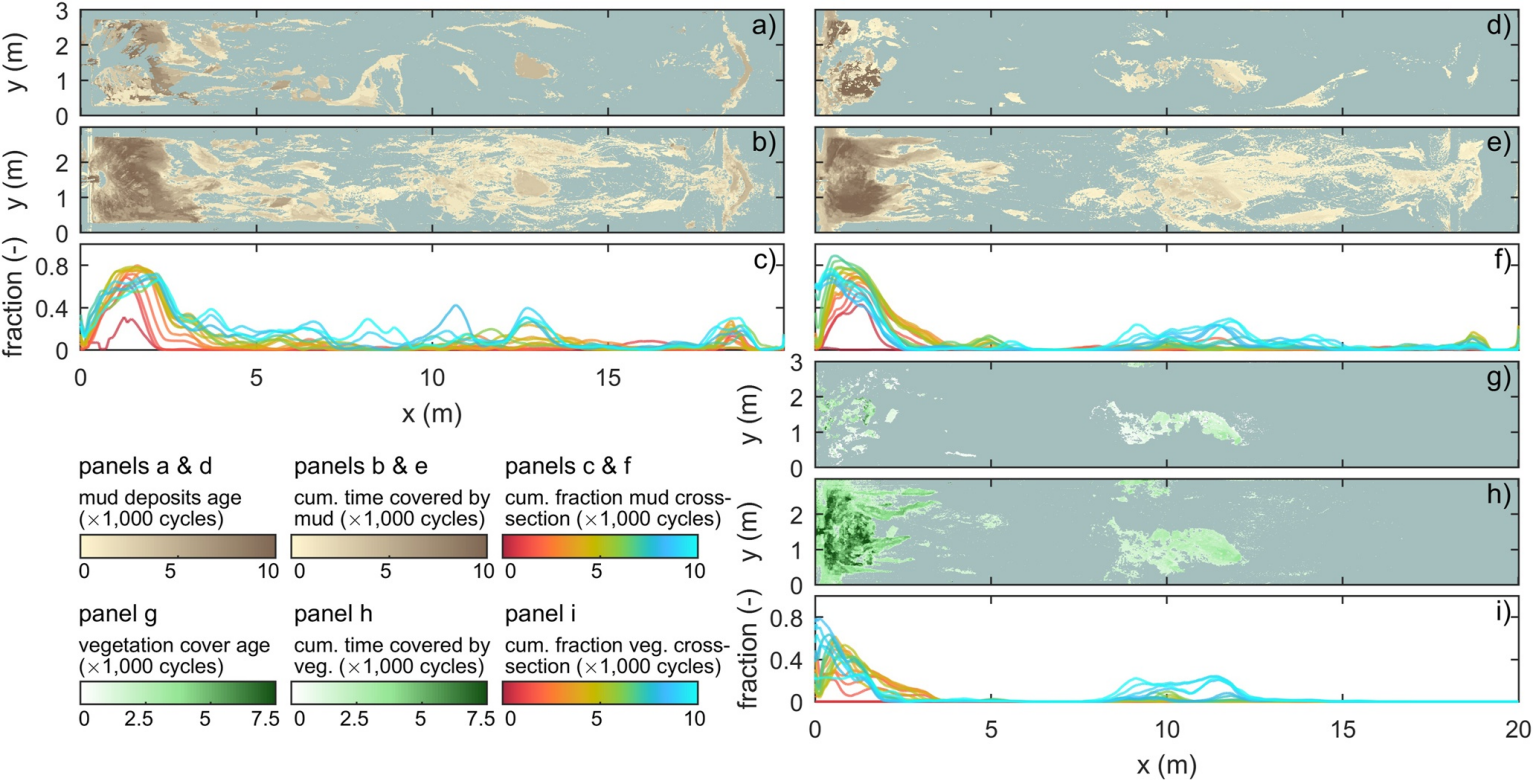
Mud and vegetation distribution throughout the second experiment with mud (left column) and the third experiment with mud and vegetation (right column). (a and d) Age of mud deposits at the surface at the end of the experiment. (b and e) Cumulative time over which the bed was covered with mud during the experiment. (c and f) Fraction over the cross‐section that was covered with mud along the estuary over time. (g) Age of vegetation at the end of the experiment. (h) Cumulative time over which the bed was covered with vegetation during the experiment. (i) Fraction over the cross‐section that was covered with vegetation along the estuary over time.

Fluvial mud was mainly deposited on the bay‐head delta in the first 3,500 tidal cycles (Figures 5b and 6a–6f), resulting in a higher and more prograded delta than in the sand‐only experiment (Figure 4). A small fraction of the mud was transported off the bay‐head delta and transported to low‐dynamic parts of the delta toe by tidal flows. Mud deposits were partly eroded by channel avulsion and overtopped by new sand and mud, resulting in multiple mud layers in the subsurface of the bay‐head delta that were thicker, more abundant and more laterally continuous further away from the long axis of the flume (see Text S4 in Supporting Information S1). After 3,500 tidal cycles, landward migrating tidal channels reached and reworked part of the fluvial mud which was then transported to form intertidal mudflats mainly in the upstream half of the estuary (Figures 5b–5d and 6a–6c). Meanwhile, the increased mud cover limited lateral migration of upstream channels compared to the sand‐only experiment (see Movies S1–S3).

**Figure 7:**
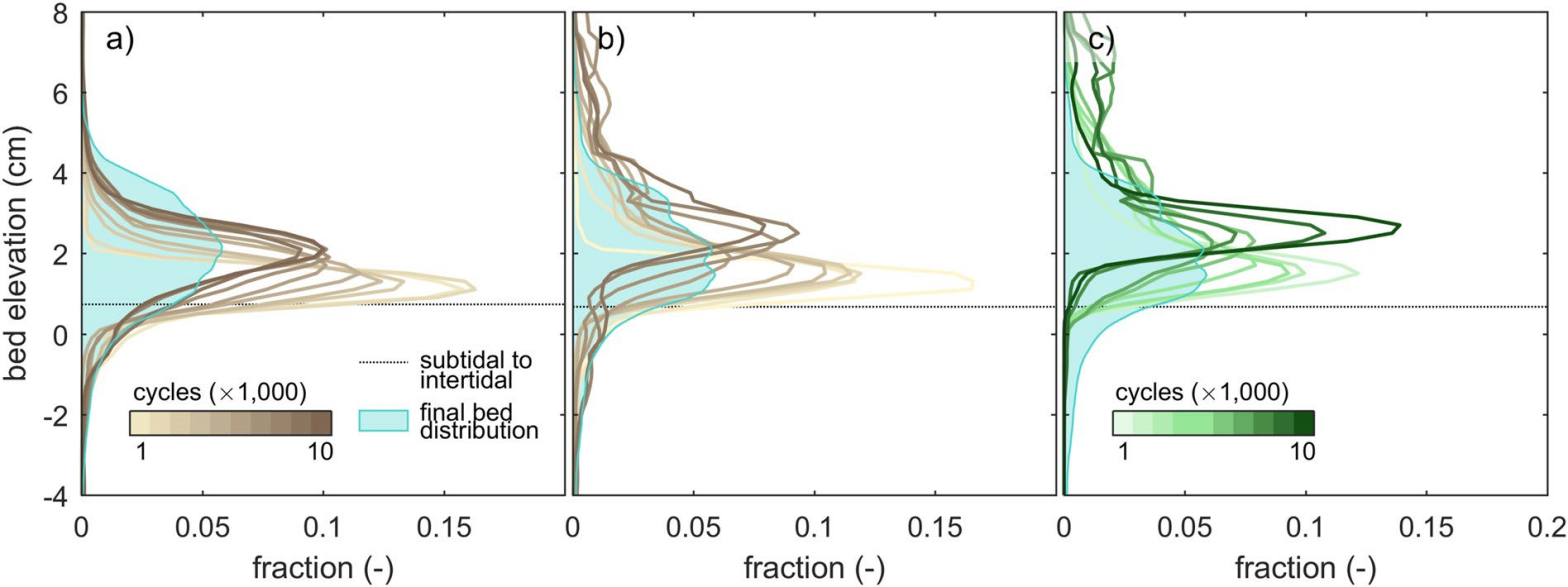
Mud and vegetation distribution as function of bed elevation over time in comparison to the bed level distribution at the end of the experiments. (a) The vertical distribution of mud in the experiment with mud and (b) in the experiment with mud and vegetation. (c) The vertical distribution of vegetation in the experiment with mud and vegetation. The boundary between subtidal and intertidal is the mean value for the entire basin.

**Figure 8:**
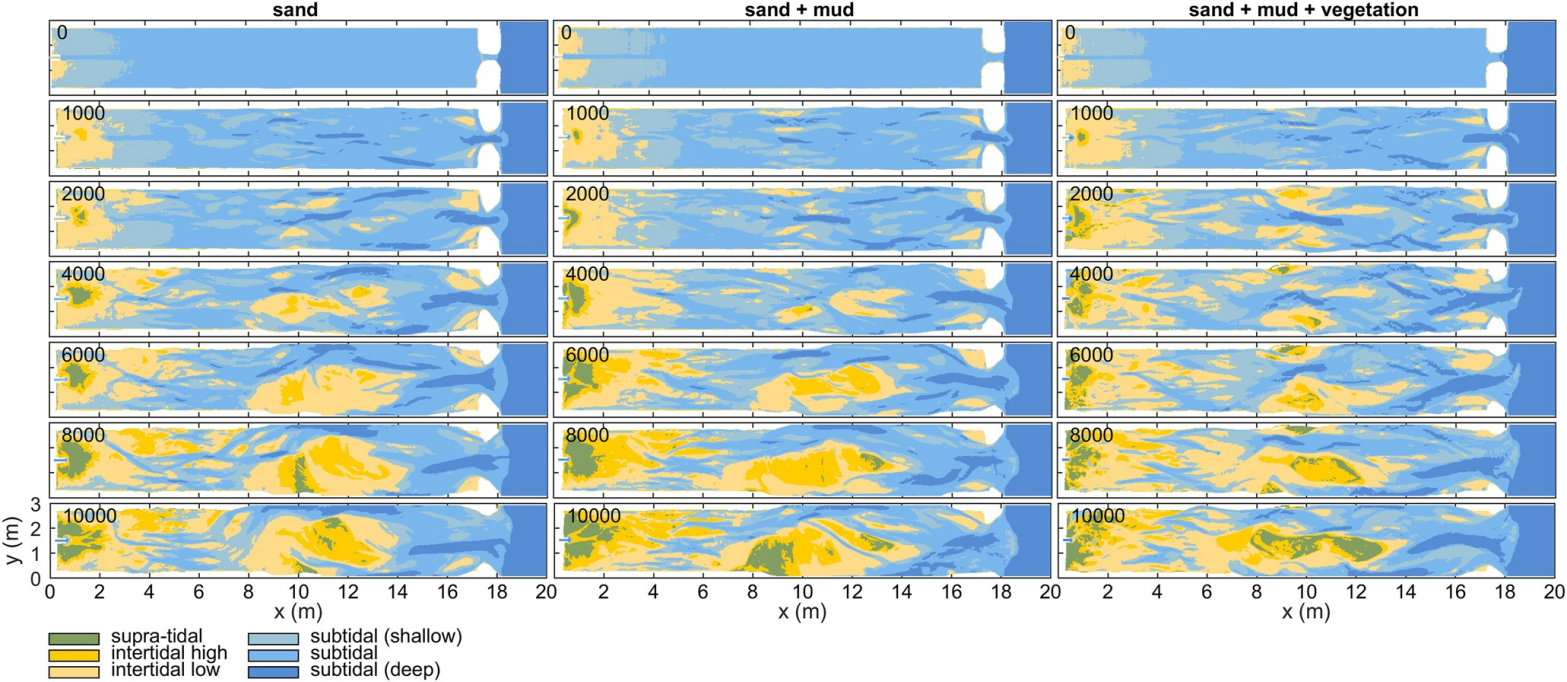
Development of subtidal, intertidal, and supratidal area of the infilling estuary experiments with only sand (left column), with mud (middle column), and with mud and vegetation (right column). Numbers indicate the tidal cycles.

Marine mud was transported partly into the estuary and partly out to sea to be deposited at the toe of the ebb‐tidal delta. Very fine marine mud was swiftly transported in the landward direction and formed small mudflats in the upstream half of the estuary within the first 1,500 tidal cycles. Coarser mud (i.e., larger than about 1 mm) moved around subtidal shoals with net little transport in the landward direction. Occasionally, this mud was trapped at the deep lee sides of subtidal bars and overridden by bar migration. Over time, many sandy tidal bars aggraded to intertidal elevations, which created new and higher sites for mudflat deposition (Figure 7a); it took until 6,000 tidal cycles to reach this stage in the downstream half of the estuary (Figures 5a and 8). Repeated visual inspection of movies (see Movies S1–S3) showed that most mudflats were completely reworked in about 2,000 tidal cycles, except for the mudflat at *x* = 13 m right between two confluences that had a longer lifespan of 3,000 tidal cycles (Figure 5f). Mudflat reworking was slower than tidal bar erosion in the sand‐only experiment and proceeded through small‐scale erosive channels at their fringes (Figure 5e), cross‐cutting channels and large‐scale channel migration. The re‐mobilized mud led to new mudflats or was transported further into the estuary. Accordingly, the upstream half of the estuary became progressively muddier, with numerous mud‐covered bars and mudflats merging with the bay‐head delta (Figures 5b, 6a–6c and 8).

### 3.3 Vegetation Patterns

Vegetation settling started on the low‐energetic parts of the bay‐head delta and on elongated shore‐connected mudflats flanking tidal channels. As fluvial mud and plants increasingly focused flow into the channels, the light *Veronica* seeds were transported further into the estuary, from where they dispersed to low‐energetic zones near the bay‐head delta. Additionally, the increased flow led to the development of more shoals on the landward end of tidal channels near the bay‐head delta.

Fluvial mud and vegetation effectively filled the low‐energetic region between the bay‐head delta and the tidal channels, leaving one open channel of about 5 cm wide as a connection between the river inflow and the tide‐dominated reach (Figure 5i). Muddy, vegetated levees formed along this channel with predominantly *Medicago* and *Lotus*, whilst the sandy channel bed was slowly aggrading. The surrounding low‐lying areas, here interpreted as fluvial floodplains, were covered with vegetation of all three species, with slightly more *Veronica* in the downstream direction. Given the increasing superposition of the channel above the surrounding landscape on the bay‐head delta, crevasse splays occurred intermittently (Figure 5i). Yet, few became successful avulsions due to the dense vegetation and absence of flood discharges, and most splay channels were backfilled with mud and covered by sparse vegetation. Distal floodplains often had deep stagnant water (i.e., >0.5 cm), which in nature could facilitate peat formation. However, the selected species were unsuitable for peat formation and instead drowned and decayed (Figure 5i), with some of the resultant organic matter being eroded later. The combination of reduced avulsion and local filling of accommodation space on the floodplains resulted in a more irregular bay‐head delta shape than in the non‐vegetated experiments.

As high‐intertidal bars formed in the estuary after 2,000 tidal cycles (Figure 8), they were sparsely colonized by *Veronica* and fewer *Lotus*, both mainly of marine origin. The vegetation sprouted on both muddy and sandy parts. *Veronica* usually established over a larger vertical range and ended up highest on the bars due to its smaller seed size. The vegetation sheltered regions that captured mud (Figures 7b and 7c) and new seeds but little sand. However, no clear order was found in the establishment of mudflats vegetation, as one could lead to the other, and vice versa in other places. The first pioneering vegetation on bars was located close to the banks, where it first steered the flow to cause considerable erosion of the non‐vegetated outer banks, after which the bars either merged with the outer banks or were eroded by migrating channels.

Expanding tidal channels eroded and sandy bars buried the vegetated mudflats near the bay‐head delta (around *x* = 3 m), strongly reducing the vegetation coverage after 5,000 tidal cycles. Fluvial sediment did not reach this area but was mainly trapped on the bay‐head delta, which strongly grew in elevation compared to the non‐vegetated experiments (Figure 4). Most eroded vegetation was simply uprooted but some was preserved as layers of organic matter in the subsurface. Repeated visual inspection of movies showed that most muddy and sandy bars in the upstream half of the estuary were reworked within 1,000 tidal cycles, while only intertidal bars close to the vegetated bay‐head delta covered by *Lotus* or *Medicago* persisted longer. As a result, vegetation was concentrated on the bay‐head delta and small bars around *x* = 10 m during the first 6,000 tidal cycles (Figure 6i).

Around 6,000 tidal cycles, vegetation started to establish on the large mid‐channel bar in the central estuary that also formed in the non‐vegetated experiments. At first, a small vegetated mudflat formed atop the bar that was in between the two main channels. Flood currents, particularly in the channel at *y* = 0.5 m, brought sediment to the landward side of the mid‐channel bar where it was deposited as a shoal coalescing with the mid‐channel bar (see 6,000–10,000 cycle panels in Figure 3). This effectively increased bed elevations landward of the vegetated mudflat and created a new site for establishment, first by mudflats and *Veronica* and later also by *Lotus* and some *Medicago* (Figures 5g and 5h). The vegetation inhibited flow and incision across the mid‐channel bar and caused stronger tidal currents and deeper channels parallel to the bar. This resulted in the following three developments. First, part of the mid‐channel bar remained dry and was effectively turned into new supratidal land that narrowed the estuary (Figure 8). Second, sediment was more efficiently transported through the main channels, which resulted in more landward expansion of the mid‐channel bar than in the experiments without vegetation (Figure 5k), as well as further infilling of the upstream range *x* = 3–6 m (Figure 4c). Third, the stronger currents started to steepen and erode the banks of the vegetated mid‐channel bar (Figure 5j). Seaward expansion of the bar was slower and commenced through the deposition of ridges covered by marine vegetation (*Veronica* and *Lotus*), followed by a mudflat on the back of the vegetated ridge once the bed had aggraded to high‐intertidal levels. Near the end of the experiment, the water level difference between the ebb‐ and flood‐dominated channels flanking the mid‐channel bar had become so large that mud and vegetation near the middle of the bar were eroded and an incipient cutoff channel developed (Figure 5k).

### 3.4 Infilling and Development of a Funnel‐Like Shape

**Figure 9:**
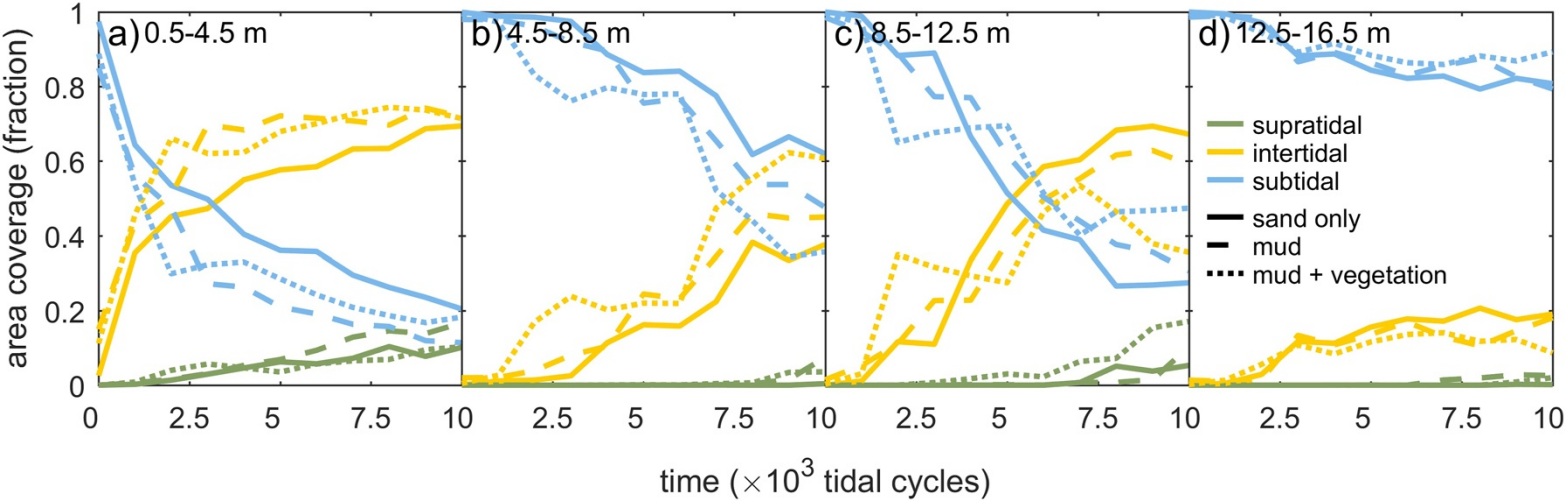
Subtidal, intertidal and supratidal area in four reaches of the estuary: (a) 0.5–4.5 m, (b) 4.5–8.5 m, (c) 8.5–12.5 m, and (d) 12.5–16.5 m.

The intertidal and supratidal areas increased in all three experiments, showing a trend of estuary infilling (Figures 8 and 9). Mud and vegetation formed more intertidal area in the upstream half of the estuary, partly because more sediment was added, and caused faster development of supratidal areas (Figures 9a–9c). On the long vegetated bar (Figure 5k), this development was at the cost of local intertidal area due to the deep, steeply sloped channels next to the vegetated bar (Figure 9c). However, as these deep channels proved very efficient in transporting sediment in the landward direction, the vegetated bar indirectly contributed to the accretion of more intertidal area in the upstream part of the estuary (Figure 9b). Mudflats were typically found in regions classified as intertidal and supratidal, whilst vegetation predominantly occupied supratidal areas (Figure 7).

**Figure 10:**
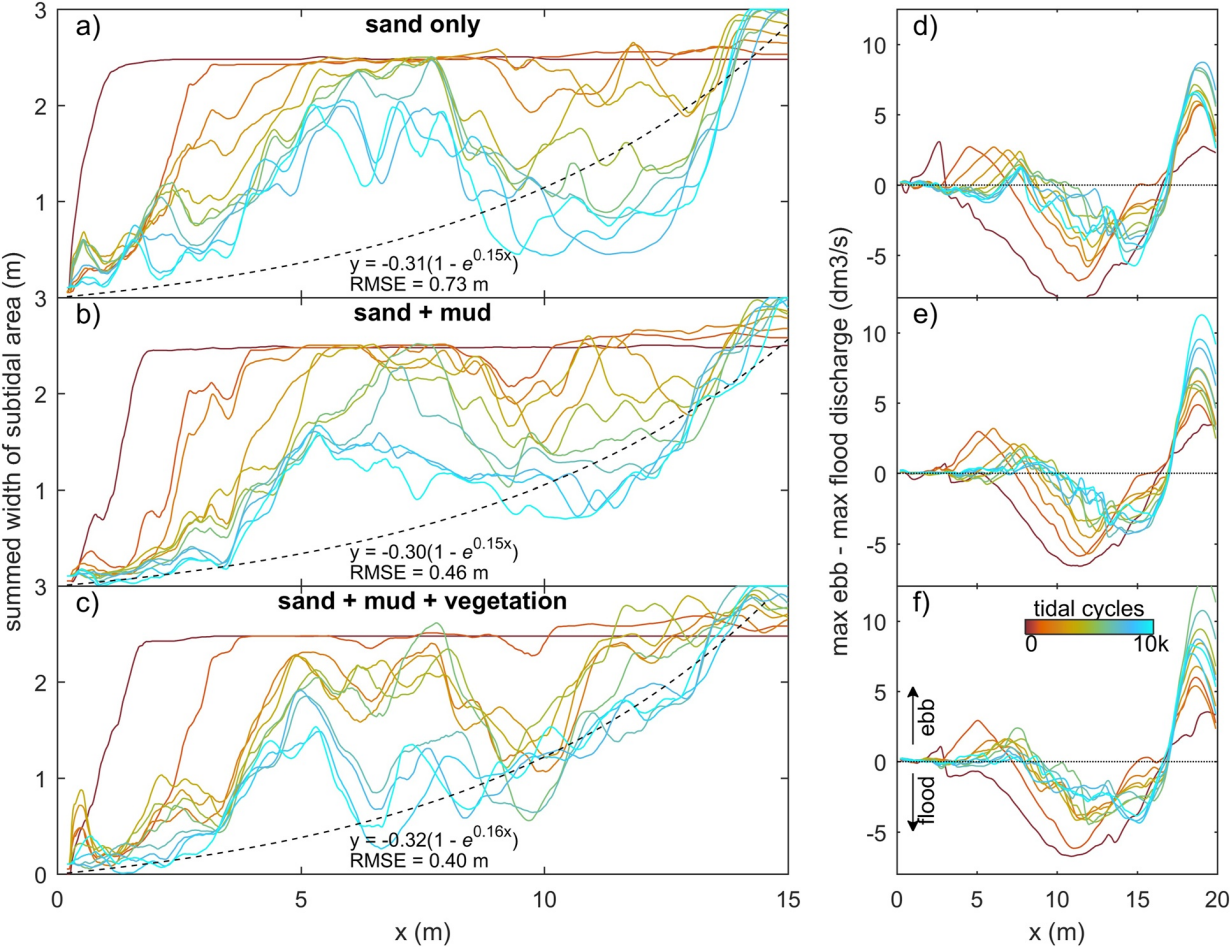
Summed width of subtidal area, showing the development toward a funnel‐shaped estuary with exponentially converging width in especially the experiment with mud and vegetation. (a) The experiment with sand only, (b) sand and mud, and (c) sand, mud, and vegetation. (d–f) Ebb or flood‐dominance along the estuary over time. As subtidal area decreases over time, the experiments become less flood‐dominant from the landward boundary.

Surprisingly, the rate of infilling in the 4.5–8.5 m reach was lower than in the seaward direction and on the bay‐head delta upstream (compare Figure 9b with Figures 9a, 9c and 9d). Here, the subtidal and intertidal areas changed linearly over time, whilst the other reaches appeared to develop quicker toward a filled, steady state. This differentiation suggests that the coastal sediment supply and tidal currents mostly caused the infilling of the seaward half of the estuary, whilst the effects of fluvial input were limited to the bay‐head delta. This is consistent with the along‐channel development in flow asymmetry (Figures 10d–10f), which rapidly became mildly ebb‐dominant on the bay‐head delta, whilst the middle estuary remained flood‐dominant throughout the experiments. At the tidal inlet, ebb‐dominance increased rapidly with the progressive infilling of the estuary.

**Figure 11:**
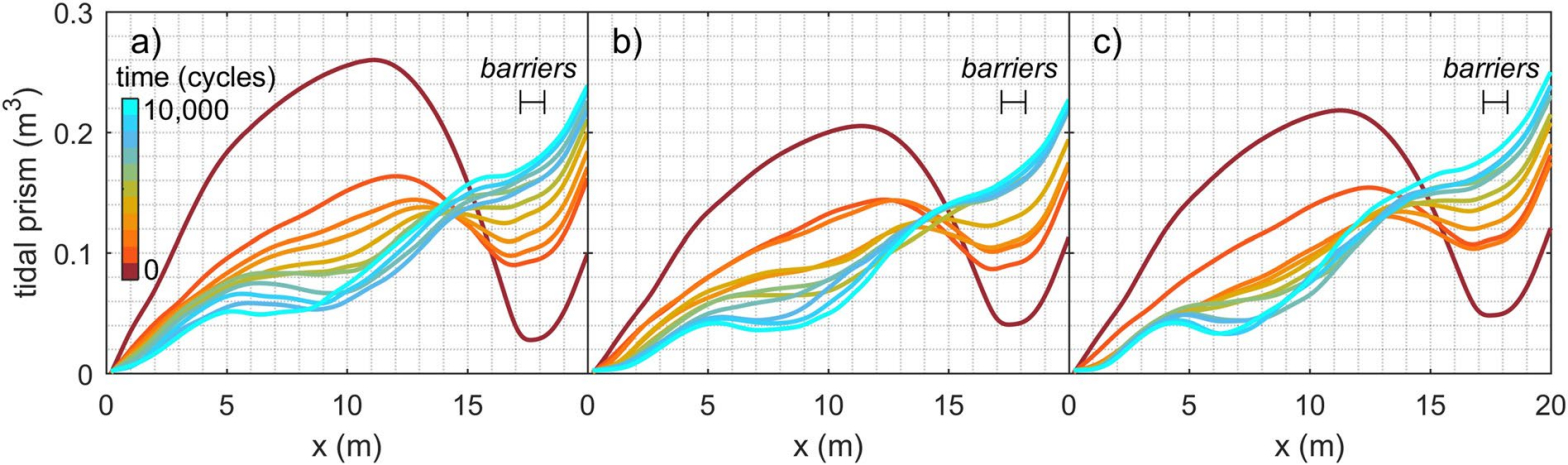
Tidal prism development along the estuary for the experiment (a) with sand only, (b) sand and mud, and (c) sand, mud, and vegetation. Differences between the initial profiles of tidal prism were due to a minor inaccuracy of the initial inlet width (±3 cm difference between experiments) but had little effect on the long‐term development.

**Figure 12:**
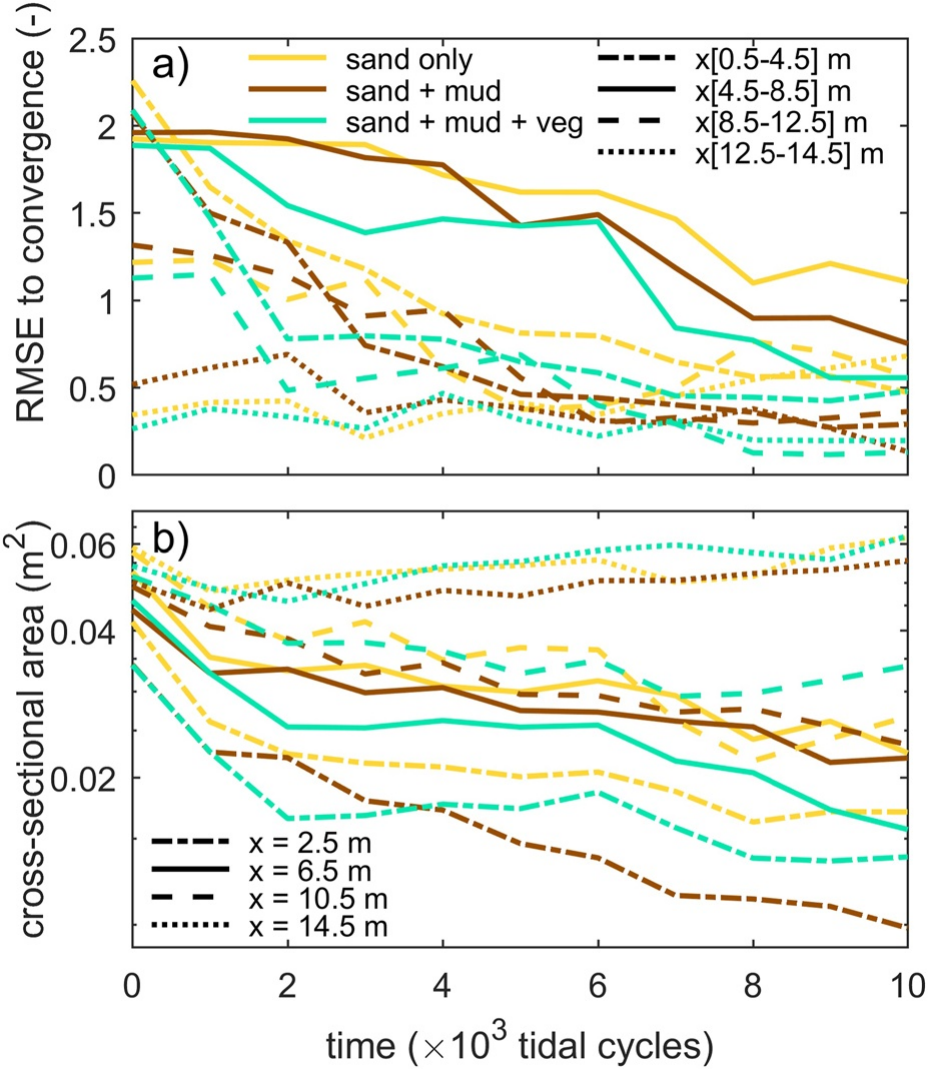
(a) RMSE of summed width of subtidal area to the convergence in Figures 10a–10c for four reaches along the estuary shows a quicker approach to subtidal channel convergence in the experiments with mud and with mud and vegetation. (b) Cross‐sectional area decreases more strongly toward the upstream boundary that is indicative of estuary infilling toward a convergent planform.

**Figure 13:**
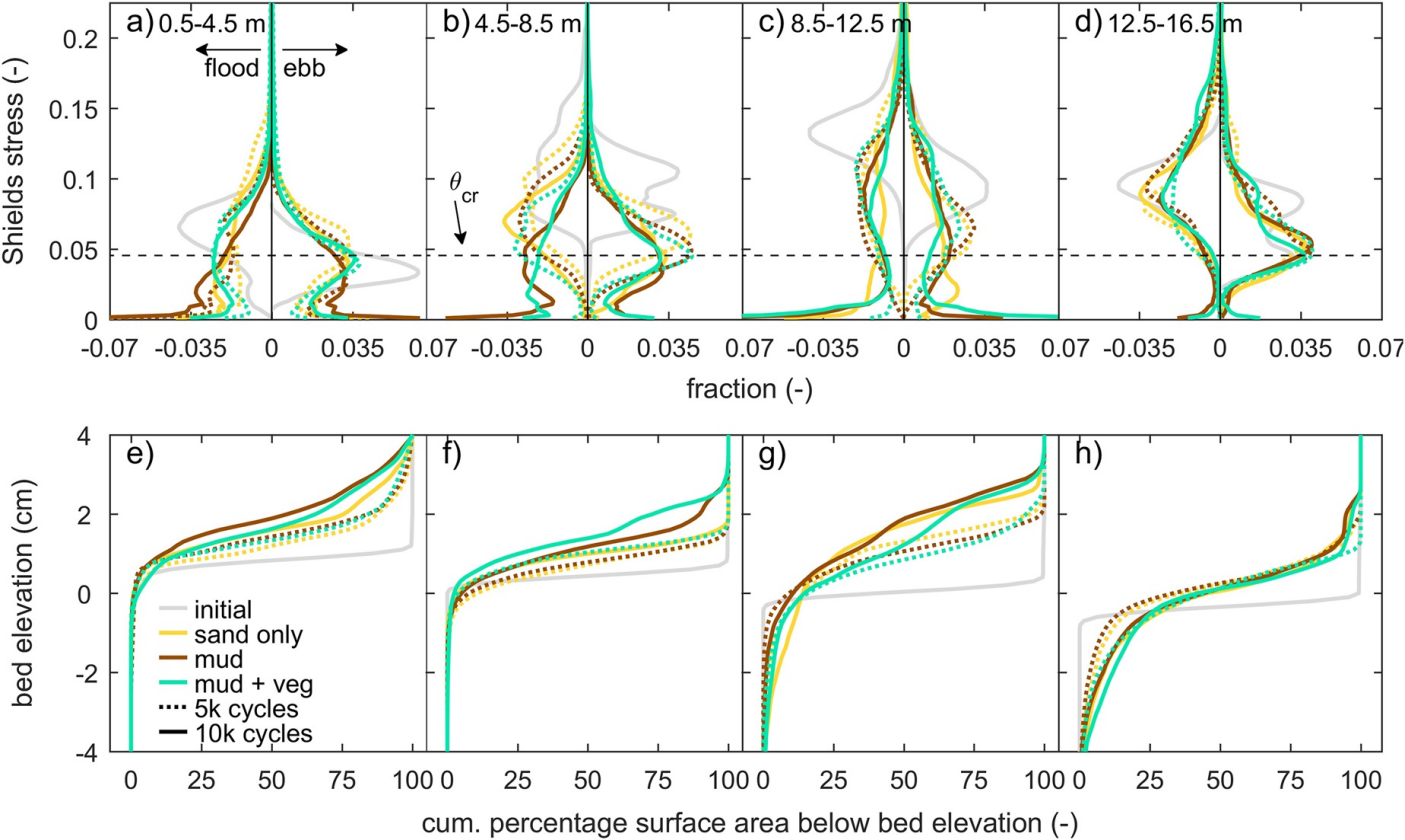
The distribution of maximum Shields stress in the flood and ebb direction, initially and at 5,000 and 10,000 cycles, split into four regions along the estuary: (a) 0.5–4.5 m, (b) 4.5–8.5 m, (c) 8.5–12.5 m, and (d) 12.5–16.5 m. The critical shields number (*θ*_cr_) for beginning of sand motion is indicated as a horizontal dashed line. For the same reaches and times, the hypsometry development is given in panels (e–h).

As intertidal and supratidal areas formed and expanded, the summed cross‐sectional width of the subtidal channels approached a convergent funnel‐like shape, especially in the experiments with mud and with mud and vegetation (Figures 10 and 12a). Figure 12a indicates that the addition of vegetation led to a temporarily faster development toward a convergent shape especially during phases of expanding vegetation patches, that is, between 2,000 and 4,000 cycles and after 6,000 cycles. The funnel‐like shape was also evident from the linear‐to‐exponential decrease in tidal prism in the landward direction (Figure 11). At the beginning of the experiments, the tidal prism increased in the upstream direction away from the tidal inlet, caused by a secondary tidal wave in the initially deep basin driven by the tilting. Minor differences in initial inlet width caused the difference in the initial profile of tidal prism between the experiments (Figure 11). This effect quickly reduced over time as the inlet widened and the eroded sediment was captured in the estuary, which decreased sediment mobility (Figures 13a–13d) and cross‐sectional area (Figure 12b) in a morphodynamic feedback.

Sediment mobility further decreased as the experiments progressed, which is consistent with the observed estuary infilling (Figure 13). This decrease in mobility was most prominent in the mud experiment. The vegetation experiment showed a decrease in mobility toward 5,000 cycles as also seen in the other experiments, but, interestingly, this was followed by a small increase in surface area with channels with high sediment mobilities (*θ* > 0.1) toward 10,000 cycles especially in the ebb direction (Figures 13b and 13c). This trend is indicative of increased tidal penetration co‐occurring with the flow confinement along the long supratidal bar.

**Figure 14:**
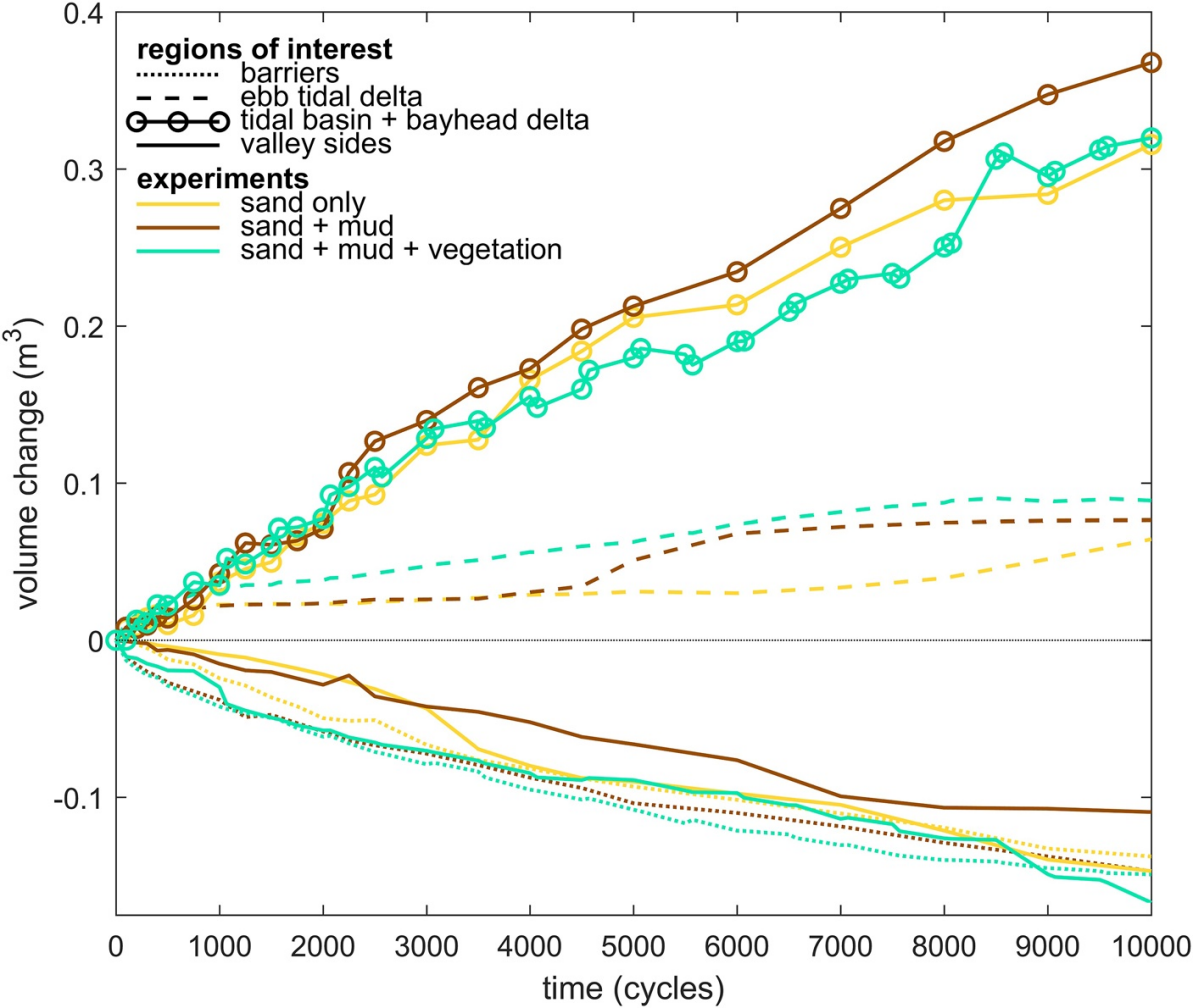
Sediment sources and sinks for the three experiments, split into four regions of interest: the barriers, the ebb‐tidal delta, the estuary plus the bay‐head delta, and the valley sides that eroded over time.

Apart from the fluvial sand input, most of the infilling resulted from reworking sediment already present in the initial bed. This sediment was first redistributed from the higher to the lower parts (Figure 14). Second, sediment was moved upstream from the barriers, of which the erosion rate was comparable between the three experiments. The valley sides were least eroded in the experiment with mud. The sediment input by the river and from the eroded valley sides and barriers was about the same as the sediment gain in the estuary and the ebb‐tidal delta. Overall, more sediment was deposited within the estuary in the mud experiment (Figure 14) and this resulted in the most infilled estuary (see also Figure 4).

## 4 Discussion

The general development in all three experiments is the infilling of a subtidal, unfilled estuary with sand, mud and even a minor amount of organic material. The experimental control on mud supply and vegetation allows for the analysis of mud and vegetation effects on the infilling trend and the morphodynamics. A generalization to natural estuaries is followed by a conceptual model for steady states at varying degrees of infilling of wave‐dominated and mixed‐energy estuaries.

### 4.1 Mud and Vegetation Effects on Estuary Infilling

Mud promoted estuary infilling because it filled accommodation space from the landward boundary, which reduced the tidal prism in the upstream half of the estuary. This can be partly ascribed to mud being added as extra sediment. However, the low density and slightly cohesive properties of the mud enabled bars to elevate well into supratidal levels. The latter two effects led to limited lateral migration of the ebb‐dominated tidal channels and a longer life span of the flood‐dominated intertidal bars as cutoffs reduced. Therefore, mud could further infill the low‐energetic regions of the estuary, including the tops of intertidal bars (Figure 5a and 5d–h) and topographical lows in the intertidal‐to‐supratidal range (Figures 5b, 7a and 7b). This development made the estuary progressively muddier and shallower toward the landward boundary, which is consistent with observations in nature (e.g., Baas et al., 2008; Glenn, 1978; Van Maren et al., 2016), numerical modeling (Braat et al., 2017; Lokhorst et al., 2018; Van de Lageweg et al., 2018) and previous experiments in the Metronome (Braat, Leuven, et al., 2019). Also, mud contributed to a seaward shift of the transition from flood‐dominance to (weak) ebb‐dominance downstream of the bay‐head delta (Figure 10e). Yet, mud in the experiments was generally not deposited as shore‐connected mudflats that flank many natural estuaries (e.g., Dalrymple & Choi, 2007; Dalrymple et al., 1992), which was due to strong currents along the banks of the estuary. The formation of such shore‐connected mudflats probably requires a broader range of suspended sediments (Braat, Leuven, et al., 2019) and diurnal variability or a spring‐neap cycle. This is analogous to how floods in meandering rivers create floodplains (e.g., Van Dijk et al., 2013).

Vegetation captured part of the mud, which resulted in fewer and smaller mudflats compared to experiments without vegetation (Figure 6). This process contributed to the quicker formation of supratidal areas particularly in the middle reach of the estuary and near the bay‐head delta (Figures 3 and 8). While vegetation influenced, but did not determine, where mud settled in the estuary, neither mud nor vegetation was found necessary for the settling of the other. This observation agrees with both field observations (e.g., Van der Wal et al., 2008) and recent numerical modeling with mud and with dynamic vegetation (Braat, Van Dijk, et al., 2019; Brückner et al., 2019). As emergent areas developed (Figures 13e–13h), vegetation in the experiment generally established in the supratidal range where it enhanced mud deposition (Figure 7), in qualitative agreement with field observations (e.g., Allen, 2000; Mudd et al., 2009; Temmerman et al., 2007). Additionally, vegetation focused the flow between patches as also found in rivers (e.g., Braudrick et al., 2009; Luhar et al., 2008), resulting in channel formation on the bay‐head delta (Figure 5i) and in creek formation on the vegetated bars (Figures 5g and 5h) in a similar fashion as on natural salt marshes (e.g., Temmerman et al., 2007). On the larger system‐scale, vegetation indirectly caused more landward sediment transport especially after 8,000 cycles (Figures 8a–8c, 10, 13e–13g and 14).

**Figure 15:**
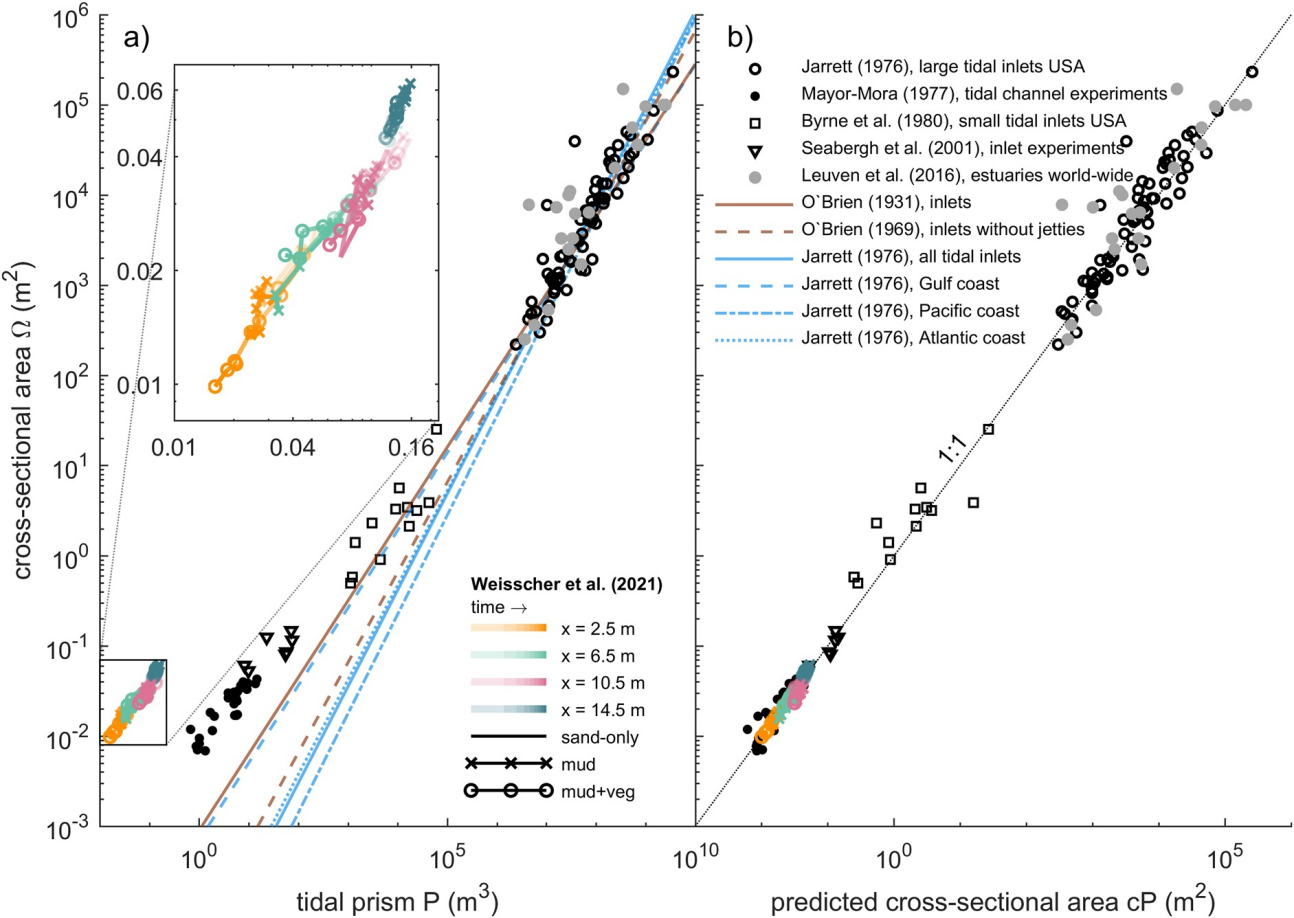
Development of cross‐sectional area versus tidal prism in comparison to natural tidal systems and previous scale experiments of tidal inlets. (a) Scale experiments deviate from the extrapolated relations that are based on natural tidal systems. The inset shows the temporal development for the three experiments of this study at four locations along the flume. (b) Cross‐sectional area plotted against predicted cross‐sectional area, which is tidal prism corrected for larger friction effects in scale experiments using *c* = *π*/(*TU*_max_) (Equations (1), (2), (3); O’Brien, 1969; Seabergh et al., 2001). The graph shows similar behavior for natural tidal systems and their down‐scaled counterparts. *Note*. Only data in the field data sets are plotted for which peak flow velocity at the tidal inlet/estuary mouth was available.

Tidal prism in the novel experiments scaled similarly to cross‐sectional area as in natural estuaries (Byrne et al., 1980; Jarrett, 1976; Leuven, De Haas, et al., 2018; O’Brien, 1969) and previous scale experiments (Mayor‐Mora, 1977; Seabergh et al., 2001; Figure 15). However, while some numerical studies (Lanzoni & Seminara, 2002; Zhou et al., 2014) and scale experiments (Stefanon et al., 2010, 2012) show that the *P*Ω relation (Equation 3) is approached in the initial phase of morphological evolution (not included in Figure 15), this initial imbalance is absent in this study. This is probably caused by the tilting mechanism, which caused an initially large tidal prism within the basin that was larger than expected from the cross‐sectional area at the inlet (Figure 11). Nevertheless, the large tidal prism quickly reduced as the estuary filled with sediment eroded from the barrier, which also increased the mouth width. The inlet widening in the experiments was by design to generate unlimited marine sediment supply, as it was practically impossible to reproduce sediment exchange with the littoral zone in the experimental setup. Alternatively, the barriers could have been fixed but this would probably steer considerable bank erosion until the estuary drops below the threshold of motion. Regardless of the differences with nature, where the inlet tends to decrease as accommodation space is filled (e.g., Jarrett, 1976; O’Brien, 1931, 1969), the experimental estuaries converged.

While convergent, single‐channel estuaries can have a balanced ebb‐ and flood‐generated sediment transport (Dronkers, 2017; Savenije, 2015), many estuaries have a more complicated morphology. Recently, bar‐dominated estuaries were shown to have, on average, a convergent shape if only the cross‐sectional area of subtidal channels is considered (Leuven, Braat, et al., 2018). This is consistent with the one‐dimensional flow model concept of Friedrichs and Aubrey (1988), in which the subtidal channels convey the momentum of flow and the intertidal zone only acts as flood storage to affect water surface amplitude and ebb‐dominance. As such, a multi‐channel estuary can be convergent in the subtidal cross‐sectional channel area whilst this may not be directly apparent from the estuary morphology (Leuven, Verhoeve, et al., 2018). The new experiments show that these empirical findings extend to wide infilling basins, where mud and vegetation quicken the self‐confinement of the subtidal channels in experimental estuaries toward a funnel‐like shape (Figures 10, 12 and 15). Indeed, at the end of all three experiments, the tidal prism at the mouth (0.17–0.20 m^3^) was about similar to the final stage of previous experiments of muddy ingressive estuaries that developed by widening an initial narrow channel in the same flume (Braat, Leuven, et al., 2019). The development to such a convergent shape in an initially wide, infilling basin is also found in palaeogeographical reconstructions of systems such as the Old Rhine Estuary (NL; De Haas et al., 2018, 2019), the Oer‐IJ Estuary (NL; Vos et al., 2015) and the Shoalhaven River (AUS; Roy et al., 1980). Together with previous findings, the new experiments show that a convergent shape with mid‐channel bars can be considered an attractor state (cf. Thorn & Welford, 1994), as the subtidal channels of both ingressive and infilling estuaries tend to develop to such a shape.

The question is now to what degree the two mechanisms, filling and confining, contributed to the trend toward a multi‐channel estuary that is on average convergent. Interestingly, the mud experiment was most infilled (Figure 14), whilst the mud‐vegetation experiment had developed slightly closer to a convergent shape (Figures 10 and 12a). This convergent shape of the subtidal channels is somewhat obscured by an intricate pattern of intertidal bars and flats, compared to an ideal estuary. As these intertidal areas contribute to the tidal prism, mud and vegetation affected the subtidal channel and inlet dimensions of the whole system. At first, both mud and vegetation reduced tidal prism as overall higher bed elevations developed compared to the sand‐only experiment (Figures 4 and 13), which caused friction‐dominance over convergence effects (Dalrymple & Choi, 2007; Friedrichs, 2010). However, whilst the mud experiment continued to reduce tidal prism, the combination of mud and vegetation seemed to lead to a slight increase in tidal prism toward the end of the experiment (Figures 11c). This apparent increase opposes current understanding that vegetation reduces tidal prism (FitzGerald et al., 2008, 2018) and more experiments are needed to determine whether the apparent increase in tidal prism, as well as the slightly more convergent shape, are significant or due to internal variability or unintended vegetation dynamics of the system.

The novel experiments fill a gap in the set of previous long‐term morphodynamic modeling and experiments, which focused predominantly on ingressive estuaries (e.g., Braat, Leuven, et al., 2019; Braat et al., 2017; Leuven, Braat, et al., 2018; Van der Wegen & Roelvink, 2008) on the one hand, and on tidal basins with too little fluvial input on the other. The new experiments showed that little floodplain sediment supply (i.e., mud) leads to a wide, multi‐channel (braided) estuary, for example, similar to the Oer‐IJ Estuary (NL; Vos et al., 2015). On the other hand, ample floodplain sediment supply contributed to a narrow estuary with levees and high floodplains that is closer to the classic, ideally converging estuary (Savenije, 2015). Furthermore, the experiments indicated a disconnect between the effects originating from the fluvial and marine boundary conditions. Specifically, the development of sub‐, inter‐, and supratidal areas (Figures 9 and 12a) indicated that the bay‐head delta reach and the downstream half of the estuary quickly approached a steady state, in contrast to the reach 4.5–8.5 m. It is the latter reach where the combined effects of the river and the tide on sediment supply (Figure 11) are smallest and the morphological response to boundary conditions takes longest, as in the unfilled bay reach in the conceptual model of Dalrymple et al. (1992). Thus, as most reaches were at, or close to, a steady state (Figure 12a), the experimental estuaries as a whole are considered close to a steady state as well.

### 4.2 A Conceptual Model for Steady State Morphology of Estuaries

“Equilibrium” and “steady state” are contested concepts in geology (Pierik, 2021; Thorn & Welford, 1994; Zhou et al., 2017), but are useful in the context of experiments and models with controlled boundary conditions. On the geological timescale of sea‐level fluctuations, estuaries are considered ephemeral systems (Dalrymple & Choi, 2007; De Haas et al., 2018; Lanzoni & Seminara, 2002). However, on the centennial timescale, sandy estuaries have been argued to have two steady states given reasonably constant boundary conditions. The first is an unfilled estuary that is below the threshold of motion (Dalrymple et al., 1992; Roy et al., 1980), and the second is a filled estuary with bars and net converging channels. Depending on initial conditions such as valley dimensions, a filled estuary can be either a wide system with many channels or a narrower system with few channels and bars (Leuven, Braat, et al., 2018). Now, the new set of experiments suggests that also partly filled estuaries may attain a steady state.

**Figure 16:**
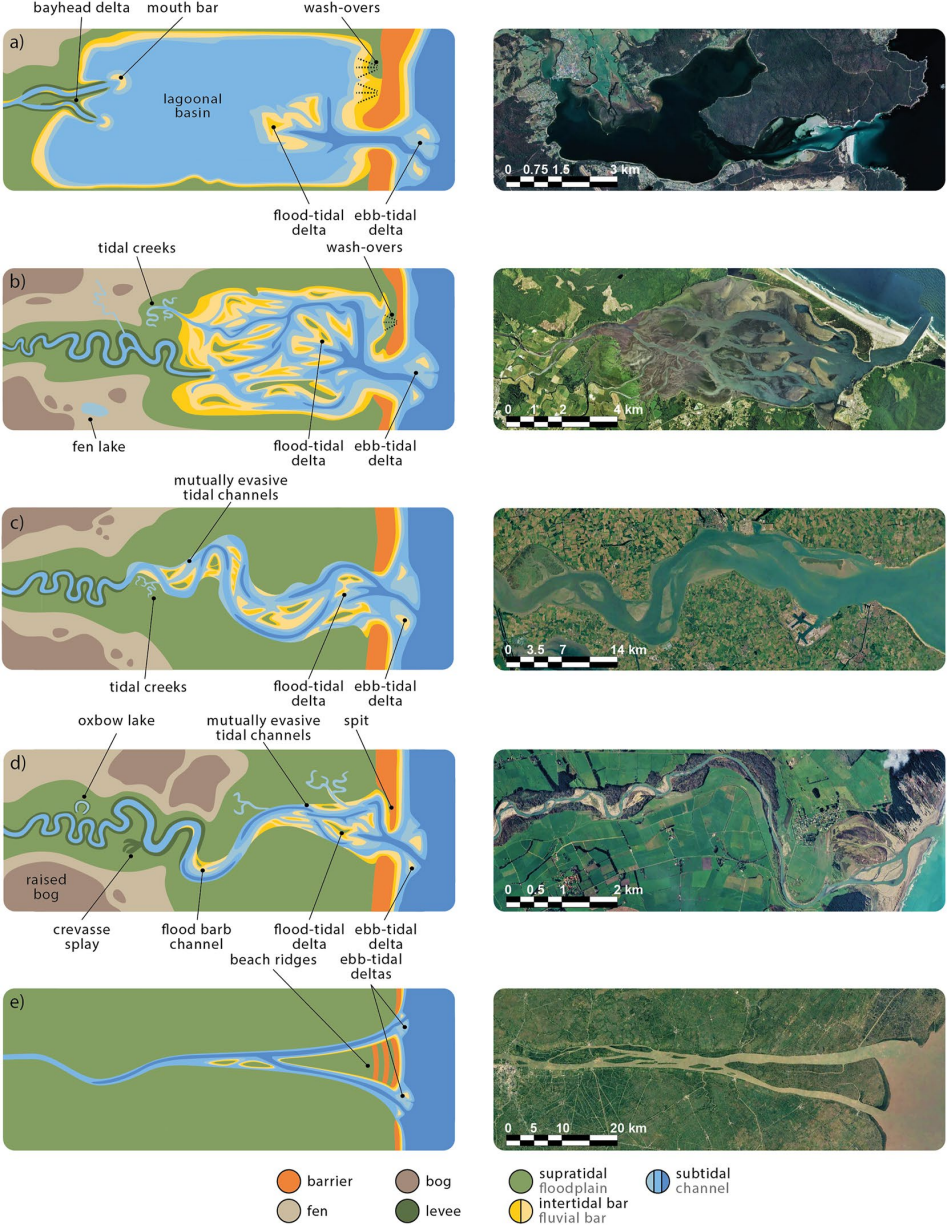
Conceptual models of (a and e) the two classic steady‐state estuaries end‐members between an unfilled estuary (after Dalrymple et al., 1992) and an ideal estuary (after Savenije, 2015) and (b–d) three examples of partly filled steady states with an increasing tendency to form floodplains, each with a natural estuary example in the right column. (a) The classic unfilled steady‐state estuary. A natural example is the Georges Bay (AUS). (b) A partly filled steady state with a large number of tidal bars and channels and may be bound by salt marshes and peatlands, such as the Tillamook Bay Estuary (USA). (c) A partly filled steady state that is more confined than (b) with on average two tidal channels, as found for instance for the Western Scheldt (NL) (Leuven, Braat, et al., 2018). (d) A partly filled steady state that is strongly confined and quickly reduces to a single channel in the upstream direction. A natural example is the Tangimoana River (NZ). (e) The classic infilled steady‐state estuary (Dronkers, 2017; Savenije, 2015). A natural example is the individual branches of the Mekong River (VT). Satellite imagery was retrieved from Google Earth.

The key concept that seems to determine to which steady state an estuary develops is the tendency to form floodplains, which is a function of variables including floodplain sediment supply, flood magnitude and frequency, tides, waves, and vegetation. Whilst the novel experiments all showed a fairly similar steady state, it is hypothesized that, for example, a higher floodplain sediment supply and more flood‐resilient vegetation would probably lead to a narrower steady state. As a result, the different tendency for floodplain formation between estuaries allows for a continuum of steady states in terms of the channel planform, convergence, and the degree of filling under reasonably constant boundary conditions on the centennial timescale. Here, the unfilled estuary and the ideal estuary are end‐members on the continuum rather than the only two alternative steady states, which is presented in a new conceptual model (Figure 16). In this model, the experiments of this study are somewhere between the Tillamook Bay Estuary (Figure 16b) and Western Scheldt Estuary (Figure 16c).

The possibility of many steady states between the conceptual unfilled estuary and the ideal estuary is consistent with the apparent steady states of some partly filled estuaries in nature. Examples include the Arcachon Bay Estuary (Figure 1c; Allard et al., 2009; Fenies & Faugères, 1998) and the Ems‐Dollard Estuary (Van Maren et al., 2016). Both systems are in a partly filled state with multiple parallel channels that remain open regardless of ample sediment supply. Similar observations are made in estuaries with mangrove vegetation, such as in Darwin Harbor (AUS; Woodroffe et al., 2016) and the Wapengo Lagoon (AUS; Nichol, 1991). Another example is the multi‐channel Western Scheldt Estuary, which has on average two tidal channels and mid‐channel bar complexes that remain largely in place due to topographic forcing by the varied width of the estuary (Figure 16c). The mechanisms leading to this steady estuary planform were demonstrated in previous Metronome experiments (Leuven, Braat, et al., 2018). On the timescale of formation of their experiments and of the Western Scheldt Estuary (Van der Spek, 1997), there was no apparent development toward a single convergent channel. Even with high mud supply, previous experimental estuaries remained multi‐channel systems (Braat, Leuven, et al., 2019). Nevertheless, the multiple channels were collectively convergent, which is consistent with the physical principles underlying the ideal estuary (Savenije, 2015).

This is not to say that any state can be stable, and that the stable width of estuaries is entirely unconstrained. Data from 68 estuaries show a surprisingly strong correlation between the width of the river upstream of the tidally influenced reach and the estuarine convergence length and the width of the mouth (Figures 4b and 4e; Leuven, van Maanen, et al., 2018). The hydraulic geometry of channels in a tidal delta systematically vary with river discharge and tidal amplitude (Sassi et al., 2012), and channel dimensions in a rapidly shifting estuary with erodible banks generally correspond to those of the upstream river (Shimozono et al., 2019). How upstream river discharge controls the dimensions of estuaries is not clear and requires further work.

The antecedent landscape (i.e., the initial conditions) and boundary conditions both determine the degree of infilling and the steady state reached. Starting from a narrow, converging (ingressed) channel, previous experimental studies (Braat, Leuven, et al., 2019; Leuven, Braat, et al., 2018) reached a multi‐channel steady state resembling Figure 16c through ingression, as opposed to the infilling cases in the present study. This contrast illustrates that the filling of estuaries is not necessarily a one‐way process but depends on the boundary conditions, including relative sea‐level, hydrodynamics, sediment supply and eco‐engineering traits of vegetation. Moreover, these opposing ways of reaching a steady state have implications for the geological record that require further study. Ingressive estuaries generally only have facies of estuary filling at their margins (e.g., Vos, 2015), whilst infilling estuaries have infilling facies ranging much further outward of their present‐day margins (e.g., Clement et al., 2017; De Haas et al., 2018; Vos, 2015).

The existence of a continuum of steady states raises the question whether large events and disturbances can cause a shift to another state. This is understudied, but published work suggests that storm and flooding events can have two contrasting impacts on estuary state. Events may set back the filling sequence in estuaries through erosion of predominantly intertidal and supratidal area (e.g., Mariotti et al., 2010) or events may temporarily increase the import of sediment derived from coastal erosion (e.g., Fruergaard et al., 2013). The effects of events and disturbances are often merely noise at the system scale and the estuary remains in its steady state; for instance, this has been shown for the Humber Estuary (UK) following a large river flood (Townend et al., 2007) and for the Firth of Thames (NZ) following storms and river floods (Swales et al., 2019). Similar findings apply at the smaller scale of salt marshes following hurricanes (FitzGerald & Hughes, 2019). In case the trigger is large enough, the resultant change in accommodation space can perhaps cause a shift toward another steady state. For example, this happened with the catastrophic formation of the Dollard tidal embayment in the Ems Estuary, the Netherlands (De Haas et al., 2018; Pierik, 2021; Van Maren et al., 2016). A persistent change in boundary conditions could also force the system to another steady state (e.g., Pierik, 2021).

## 5 Conclusions

Physical scale experiments were conducted of infilling estuaries with mud and vegetation in the tilting flume the Metronome, which resulted in the following insights. Estuaries have a continuum of steady states between the classic concepts of the unfilled and completely infilled, ideally converging estuary. Intermediate cases can develop a converging planform with multiple channels. Mud and vegetation affect the degree of filling and the development of an overall converging system through two mechanisms: accretion in bars and system margins, and lateral confinement through flow concentration in channels. The first mechanism of filling increases the intertidal and supratidal area and reduces the tidal prism. The second mechanism of confining focuses flow into channels through increased bar elevation and hydraulic roughness caused by vegetation. Mud and especially vegetation elevated bars well above the mean high water level and increased the stability and longevity of bars. Mud contributed to overall slightly higher bed elevations, particularly on the bayhead delta and in the upstream basin. Vegetation reduced the mudflat area but led to faster bed level rise well into the supratidal range. The bar top stabilization further reduced shortcut channels and bar removal. The combination of mud and vegetation proved conducive to narrowing of the wide estuary toward a convergent estuary, where the coastal and fluvial sediment supply and the plant properties determined the pacing of the confining processes.

## Supporting information

Supporting Information S1

Movie S1

Movie S2

Movie S3

## Data Availability

Additional materials are available in an online supplement and the data are available via https://doi.org/10.24416/UU01-R82TWE. The online supplement contains (S1) a hydrodynamic modeling validation with Nays2D, (S2) the main results of pilot experiments in a smaller flume, (S3) an algae and fungi protocol, (S4) photographs of cross‐sections over the bay‐head delta in the experiments with mud and with mud and vegetation, and three movies of the development of the experiments. The data package includes the DEMs acquired by stereo‐photography and laser scanning, overhead imagery of the dry bed, input data for the model Nays2D, and the raw and processed output files of the hydrodynamic modeling. Consult the data package by Weisscher et al. (2020) for installation and use of the open‐source model Nays2D.

## References

[jgrf21488-bib-0001] Allard, J. , Chaumillon, E. , & Féniès, H. (2009). A synthesis of morphological evolutions and Holocene stratigraphy of a wave‐dominated estuary: The Arcachon lagoon, SW France. Continental Shelf Research, 29(8), 957–969. 10.1016/j.csr.2008.11.017

[jgrf21488-bib-0002] Allen, J. R. (2000). Morphodynamics of Holocene salt marshes: A review sketch from the Atlantic and Southern North Sea coasts of Europe. Quaternary Science Reviews, 19(12), 1155–1231. 10.1016/s0277-3791(99)00034-7

[jgrf21488-bib-0003] Baas, J. H. , Jago, C. , Macklin, M. , & Team, C. C. C. R. (2008). The river–estuarine transition zone (RETZ) of the Afon Dyfi (West Wales) as test bed for sediment transfer between river catchments and coastal environments. In BSRG 2008.

[jgrf21488-bib-0004] Baumgardner, S. (2016). Quantifying Galloway: Fluvial, tidal and wave influence on experimental and field deltas (PhD thesis). University of Minnesota.

[jgrf21488-bib-0005] Beets, D. J. , & Van der Spek, A. J. F. (2000). The Holocene evolution of the barrier and the back‐barrier basins of Belgium and The Netherlands as a function of late Weichselian morphology, relative sea‐level rise and sediment supply. Netherlands Journal of Geosciences, 79(1), 3–16. 10.1017/s0016774600021533

[jgrf21488-bib-0006] Braat, L. , Leuven, J. R. F. W. , Lokhorst, I. R. , & Kleinhans, M. G. (2019). Effects of estuarine mudflat formation on tidal prism and large‐scale morphology in experiments. Earth Surface Processes and Landforms, 44(2), 417–432. 10.1002/esp.4504

[jgrf21488-bib-0007] Braat, L. , Van Dijk, W. M. , Pierik, H. J. , Van de Lageweg, W. , Brückner, M. , Wagner‐Cremer, F. , & Kleinhans, M. G. (2019). Tidal bar accretion by mudflat sedimentation. EarthArXiv. 10.31223/osf.io/gq9pt

[jgrf21488-bib-0008] Braat, L. , van Kessel, T. , Leuven, J. R. F. W. , & Kleinhans, M. G. (2017). Effects of mud supply on large‐scale estuary morphology and development over centuries to millennia. Earth Surface Dynamics, 5(4), 617–652. 10.5194/esurf-5-617-2017

[jgrf21488-bib-0009] Braudrick, C. A. , Dietrich, W. E. , Leverich, G. T. , & Sklar, L. S. (2009). Experimental evidence for the conditions necessary to sustain meandering in coarse‐bedded rivers. Proceedings of the National Academy of Sciences, 106(40), 16936–16941. 10.1073/pnas.0909417106 PMC2761352 19805077

[jgrf21488-bib-0010] Brückner, M. Z. M. , Schwarz, C. , Van Dijk, W. M. , Van Oorschot, M. , Douma, H. , & Kleinhans, M. G. (2019). Salt marsh establishment and eco‐engineering effects in dynamic estuaries determined by species growth and mortality. Journal of Geophysical Research: Earth Surface, 124(12), 2962–2986. 10.1029/2019jf005092

[jgrf21488-bib-0011] Byrne, R. J. , Gammisch, R. A. , & Thomas, G. R. (1980). Tidal prism‐inlet area relations for small tidal inlets. In Coastal engineering 1980 (pp. 2517–2533). 10.1061/9780872622647.151

[jgrf21488-bib-0012] Clement, A. J. H. , Fuller, I. C. , & Sloss, C. R. (2017). Facies architecture, morphostratigraphy, and sedimentary evolution of a rapidly‐infilled Holocene incised‐valley estuary: The lower Manawatu valley, North Island New Zealand. Marine Geology, 390, 214–233. 10.1016/j.margeo.2017.06.011

[jgrf21488-bib-0013] D’Alpaos, A. , Lanzoni, S. , Marani, M. , & Rinaldo, A. (2009). On the O’Brien–Jarrett–Marchi law. Rendiconti Lincei, 20(3), 225–236. 10.1007/s12210-009-0052-x

[jgrf21488-bib-0014] Dalrymple, R. W. , Boyd, R. , & Zaitlin, B. A. (1994). History of research, types and internal organisation of incised‐valley systems: Introduction to the volume. SEPM Society for Sedimentary Geology, 51, 1–10. 10.2110/pec.94.12.0003

[jgrf21488-bib-0015] Dalrymple, R. W. , & Choi, K. (2007). Morphologic and facies trends through the fluvial–marine transition in tide‐dominated depositional systems: A schematic framework for environmental and sequence‐stratigraphic interpretation. Earth‐Science Reviews, 81(3–4), 135–174. 10.1016/j.earscirev.2006.10.002

[jgrf21488-bib-0016] Dalrymple, R. W. , Zaitlin, B. A. , & Boyd, R. (1992). Estuarine facies models; conceptual basis and stratigraphic implications. Journal of Sedimentary Research, 62(6), 1130–1146. 10.1306/d4267a69-2b26-11d7-8648000102c1865d

[jgrf21488-bib-0017] De Haas, T. , Pierik, H. J. , Van der Spek, A. J. F. , Cohen, K. M. , Van Maanen, B. , & Kleinhans, M. G. (2018). Holocene evolution of tidal systems in The Netherlands: Effects of rivers, coastal boundary conditions, eco‐engineering species, inherited relief and human interference. Earth‐Science Reviews, 177, 139–163. 10.1016/j.earscirev.2017.10.006

[jgrf21488-bib-0018] De Haas, T. , Van der Valk, L. , Cohen, K. M. , Pierik, H. J. , Weisscher, S. A. H. , Hijma, M. P. , et al. (2019). Long‐term evolution of the Old Rhine estuary: Unravelling effects of changing boundary conditions and inherited landscape. The Depositional Record, 5(1), 84–108. 10.1002/dep2.56 31543980 PMC6743690

[jgrf21488-bib-0019] Dronkers, J. (1986). Tidal asymmetry and estuarine morphology. Netherlands Journal of Sea Research, 20(2–3), 117–131. 10.1016/0077-7579(86)90036-0

[jgrf21488-bib-0020] Dronkers, J. (2017). Convergence of estuarine channels. Continental Shelf Research, 144, 120–133. 10.1016/j.csr.2017.06.012

[jgrf21488-bib-0021] Fenies, H. , & Faugères, J.‐C. (1998). Facies and geometry of tidal channel‐fill deposits (Arcachon lagoon, SW France). Marine Geology, 150(1–4), 131–148. 10.1016/s0025-3227(98)00049-8

[jgrf21488-bib-0022] FitzGerald, D. M. , Fenster, M. S. , Argow, B. A. , & Buynevich, I. V. (2008). Coastal impacts due to sea‐level rise. Annual Review of Earth and Planetary Sciences, 36, 601–647. 10.1146/annurev.earth.35.031306.140139

[jgrf21488-bib-0023] FitzGerald, D. M. , Hein, C. J. , Hughes, Z. , Kulp, M. , Georgiou, I. , & Miner, M. (2018). Runaway barrier island transgression concept: Global case studies. In Barrier dynamics and response to changing climate (pp. 3–56). Springer. 10.1007/978-3-319-68086-6_1

[jgrf21488-bib-0024] FitzGerald, D. M. , & Hughes, Z. (2019). Marsh processes and their response to climate change and sea‐level rise. Annual Review of Earth and Planetary Sciences, 47, 481–517. 10.1146/annurev-earth-082517-010255

[jgrf21488-bib-0025] Fortunato, A. B. , & Oliveira, A. (2005). Influence of intertidal flats on tidal asymmetry. Journal of Coastal Research, 21(5), 1062–1067. 10.2112/03-0089.1

[jgrf21488-bib-0026] Friedrichs, C. T. (1995). Stability shear stress and equilibrium cross‐sectional geometry of sheltered tidal channels. Journal of Coastal Research, 1062–1074.

[jgrf21488-bib-0027] Friedrichs, C. T. (2010). Barotropic tides in channelized estuaries. In Contemporary issues in estuarine physics (pp. 27–61). Cambridge University Press.

[jgrf21488-bib-0028] Friedrichs, C. T. , & Aubrey, D. G. (1988). Non‐linear tidal distortion in shallow well‐mixed estuaries: A synthesis. Estuarine. Coastal and Shelf Science, 27(5), 521–545. 10.1016/0272-7714(88)90082-0

[jgrf21488-bib-0029] Fruergaard, M. , Andersen, T. J. , Johannessen, P. N. , Nielsen, L. H. , & Pejrup, M. (2013). Major coastal impact induced by a 1000‐year storm event. Scientific Reports, 3(1), 1–7. 10.1038/srep01051

[jgrf21488-bib-0030] Glenn, J. L. (1978). Sediment sources and Holocene sedimentation history in Tillamook Bay, Oregon; data and preliminary interpretations (Technical Report). US Geological Survey. 10.3133/ofr78680

[jgrf21488-bib-0031] Gregoire, G. , Le Roy, P. , Ehrhold, A. , Jouet, G. , & Garlan, T. (2017). Control factors of Holocene sedimentary infilling in a semi‐closed tidal estuarine‐like system: The Bay of Brest (France). Marine Geology, 385, 84–100. 10.1016/j.margeo.2016.11.005

[jgrf21488-bib-0032] Huggett, R. J. (2016). Fundamentals of geomorphology. Routledge.

[jgrf21488-bib-0033] Jarrett, J. T. (1976). Tidal prism: Inlet area relationships (Vol. 3). US Army Engineer Waterways Experiment Station.

[jgrf21488-bib-0034] Kleinhans, M. G. , Leuven, J. R. F. W. , Braat, L. , & Baar, A. W. (2017). Scour holes and ripples occur below the hydraulic smooth to rough transition of movable beds. Sedimentology, 64(5), 1381–1401. 10.1111/sed.12358

[jgrf21488-bib-0035] Kleinhans, M. G. , Van Der Vegt, M. , Leuven, J. R. F. W. , Braat, L. , Markies, H. , Simmelink, A. , et al. (2017). Turning the tide: Comparison of tidal flow by periodic sea level fluctuation and by periodic bed tilting in scaled landscape experiments of estuaries. Earth Surface Dynamics, 5(4), 731–756. 10.5194/esurf-5-731-2017

[jgrf21488-bib-0036] Kleinhans, M. G. , Van Dijk, W. M. , Van de Lageweg, W. I. , Hoyal, D. C. J. D. , Markies, H. , Van Maarseveen, M. , et al. (2014). Quantifiable effectiveness of experimental scaling of river‐ and delta morphodynamics and stratigraphy. Earth‐Science Reviews, 133, 43–61. 10.1016/j.earscirev.2014.03.001

[jgrf21488-bib-0037] Kleinhans, M. G. , Van Rosmalen, T. M. , Roosendaal, C. , & Van der Vegt, M. (2014). Turning the tide: Mutually evasive ebb‐and flood‐dominant channels and bars in an experimental estuary. Advances in Geosciences, 39, 21–26. 10.5194/adgeo-39-21-2014

[jgrf21488-bib-0038] Kleinhans, M. G. , Van Scheltinga, R. T. , Van Der Vegt, M. , & Markies, H. (2015). Turning the tide: Growth and dynamics of a tidal basin and inlet in experiments. Journal of Geophysical Research: Earth Surface, 120(1), 95–119. 10.1002/2014jf003127

[jgrf21488-bib-0039] Lanzoni, S. , & Seminara, G. (2002). Long‐term evolution and morphodynamic equilibrium of tidal channels. Journal of Geophysical Research, 107(C1), 1–13. 10.1029/2000jc000468

[jgrf21488-bib-0040] Leuven, J. R. F. W. , Braat, L. , Van Dijk, W. M. , De Haas, T. , Van Onselen, E. P. , Ruessink, B. G. , & Kleinhans, M. G. (2018). Growing forced bars determine nonideal estuary planform. Journal of Geophysical Research: Earth Surface, 123(11), 2971–2992. 10.1029/2018jf004718

[jgrf21488-bib-0041] Leuven, J. R. F. W. , De Haas, T. , Braat, L. , & Kleinhans, M. G. (2018). Topographic forcing of tidal sandbar patterns for irregular estuary planforms. Earth Surface Processes and Landforms, 43(1), 172–186. 10.1002/esp.4166

[jgrf21488-bib-0042] Leuven, J. R. F. W. , van Maanen, B. , Lexmond, B. R. , Van der Hoek, B. V. , Spruijt, M. J. , & Kleinhans, M. G. (2018). Dimensions of fluvial‐tidal meanders: Are they disproportionally large? Geology, 46(10), 923–926. 10.1130/g45144.1

[jgrf21488-bib-0043] Leuven, J. R. F. W. , Verhoeve, S. L. , Van Dijk, W. M. , Selaković, S. , & Kleinhans, M. G. (2018). Empirical assessment tool for bathymetry, flow velocity and salinity in estuaries based on tidal amplitude and remotely‐sensed imagery. Remote Sensing, 10(12), 1–32. 10.3390/rs10121915

[jgrf21488-bib-0044] Lokhorst, I. R. , Braat, L. , Leuven, J. R. F. W. , Baar, A. W. , Van Oorschot, M. , Selaković, S. , & Kleinhans, M. G. (2018). Morphological effects of vegetation on the tidal‐fluvial transition in Holocene estuaries. Earth Surface Dynamics, 6(4), 883–901. 10.5194/esurf-6-883-2018

[jgrf21488-bib-0045] Lokhorst, I. R. , De Lange, S. I. , Van Buiten, G. , Selaković, S. , & Kleinhans, M. G. (2019). Species selection and assessment of eco‐engineering effects of seedlings for biogeomorphological landscape experiments. Earth Surface Processes and Landforms, 44(14), 2922–2935. 10.1002/esp.4702

[jgrf21488-bib-0046] Luhar, M. , Rominger, J. , & Nepf, H. (2008). Interaction between flow, transport and vegetation spatial structure. Environmental Fluid Mechanics, 8(5), 423–439. 10.1007/s10652-008-9080-9

[jgrf21488-bib-0047] Mariotti, G. , Fagherazzi, S. , Wiberg, P. L. , McGlathery, K. J. , Carniello, L. , & Defina, A. (2010). Influence of storm surges and sea level on shallow tidal basin erosive processes. Journal of Geophysical Research, 115(C11), 1–17. 10.1029/2009jc005892

[jgrf21488-bib-0048] Mayor‐Mora, R. E. (1977). Laboratory investigation of tidal inlets on sandy coasts (Technical Report). California Univ Berkeley Hydraulic Engineering Lab.

[jgrf21488-bib-0049] Moore, R. D. , Wolf, J. , Souza, A. J. , & Flint, S. S. (2009). Morphological evolution of the Dee Estuary, Eastern Irish Sea, UK: A tidal asymmetry approach. Geomorphology, 103(4), 588–596. 10.1016/j.geomorph.2008.08.003

[jgrf21488-bib-0050] Mudd, S. M. , Howell, S. M. , & Morris, J. T. (2009). Impact of dynamic feedbacks between sedimentation, sea‐level rise, and biomass production on near‐surface marsh stratigraphy and carbon accumulation. Estuarine. Coastal and Shelf Science, 82(3), 377–389. 10.1016/j.ecss.2009.01.028

[jgrf21488-bib-0051] Nichol, S. L. (1991). Zonation and sedimentology of estuarine facies in an incised valley, wave‐dominated, microtidal setting, New South Wales, Australia. In Tidal sedimentology (pp. 41–58). CSPG Special Publications.

[jgrf21488-bib-0052] O’Brien, M. P. (1931). Estuary tidal prisms related to entrance areas. Civil Engineering, 1(8), 738–739.

[jgrf21488-bib-0053] O’Brien, M. P. (1969). Equilibrium flow areas of inlets on sandy coasts. Journal of the Waterways and Harbors Division, 95(1), 43–52.

[jgrf21488-bib-0054] Olabarrieta, M. , Geyer, W. R. , Coco, G. , Friedrichs, C. T. , & Cao, Z. (2018). Effects of density‐driven flows on the long‐term morphodynamic evolution of funnel‐shaped estuaries. Journal of Geophysical Research: Earth Surface, 123(11), 2901–2924. 10.1029/2017jf004527

[jgrf21488-bib-0055] Pierik, H. J. (2021). Landscape changes and human–landscape interaction during the first millennium AD in The Netherlands. Netherlands Journal of Geosciences, 100(e11), 1–14. 10.1017/njg.2021.8

[jgrf21488-bib-0056] Pierik, H. J. , Cohen, K. M. , Vos, P. C. , Van der Spek, A. J. F. , & Stouthamer, E. (2017). Late Holocene coastal‐plain evolution of The Netherlands: The role of natural preconditions in human‐induced sea ingressions. Proceedings of the Geologists’ Association, 128(2), 180–197. 10.1016/j.pgeola.2016.12.002

[jgrf21488-bib-0057] Reynolds, O. (1889). Report of the committee appointed to investigate the action of waves and currents on the beds and foreshores of estuaries by means of working models, British Association Report. In Papers on mechanical and physical subjects (Vol. 2, pp. 380–481).

[jgrf21488-bib-0058] Roy, P. S. , Thom, B. G. , & Wright, L. D. (1980). Holocene sequences on an embayed high‐energy coast: An evolutionary model. Sedimentary Geology, 26(1–3), 1–19. 10.1016/0037-0738(80)90003-2

[jgrf21488-bib-0059] Sassi, M. G. , Hoitink, A. J. F. , De Brye, B. , & Deleersnijder, E. (2012). Downstream hydraulic geometry of a tidally influenced river delta. Journal of Geophysical Research, 117(F4). 10.1029/2012jf002448

[jgrf21488-bib-0060] Savenije, H. H. G. (2015). Prediction in ungauged estuaries: An integrated theory. Water Resources Research, 51(4), 2464–2476. 10.1002/2015wr016936

[jgrf21488-bib-0061] Seabergh, W. C. , King, D. B. , & Stephens, B. E. (2001). Tidal inlet equilibrium area experiments, inlet laboratory investigations (Vol. 1). US Army Corps of Engineers, Engineer Research and Development Center.

[jgrf21488-bib-0062] Shimozono, T. , Tajima, Y. , Akamatsu, S. , Matsuba, Y. , & Kawasaki, A. (2019). Large‐scale channel migration in the Sittang River estuary. Scientific Reports, 9(1), 1–9. 10.1038/s41598-019-46300-x 31285481 PMC6614478

[jgrf21488-bib-0063] Speer, P. E. , & Aubrey, D. G. (1985). A study of non‐linear tidal propagation in shallow inlet/estuarine systems Part II: Theory. Estuarine, Coastal and Shelf Science, 21(2), 207–224. 10.1016/0272-7714(85)90097-6

[jgrf21488-bib-0064] Stefanon, L. , Carniello, L. , D’Alpaos, A. , & Lanzoni, S. (2010). Experimental analysis of tidal network growth and development. Continental Shelf Research, 30(8), 950–962. 10.1016/j.csr.2009.08.018

[jgrf21488-bib-0065] Stefanon, L. , Carniello, L. , D’Alpaos, A. , & Rinaldo, A. (2012). Signatures of sea level changes on tidal geomorphology: Experiments on network incision and retreat. Geophysical Research Letters, 39(12), 1–6. 10.1029/2012gl051953

[jgrf21488-bib-0066] Swales, A. , Reeve, G. , Cahoon, D. R. , & Lovelock, C. (2019). Landscape evolution of a fluvial sediment‐rich *Avicennia marina* mangrove forest: Insights from seasonal and inter‐annual surface‐elevation dynamics. Ecosystems, 22(6), 1232–1255. 10.1007/s10021-018-0330-5

[jgrf21488-bib-0067] Tal, M. , & Paola, C. (2010). Effects of vegetation on channel morphodynamics: Results and insights from laboratory experiments. Earth Surface Processes and Landforms, 35(9), 1014–1028. 10.1002/esp.1908

[jgrf21488-bib-0068] Temmerman, S. , Bouma, T. J. , Van de Koppel, J. , Van der Wal, D. , De Vries, M. B. , & Herman, P. M. J. (2007). Vegetation causes channel erosion in a tidal landscape. Geology, 35(7), 631–634. 10.1130/g23502a.1

[jgrf21488-bib-0069] Thorn, C. E. , & Welford, M. R. (1994). The equilibrium concept in geomorphology. Annals of the Association of American Geographers, 84(4), 666–696. 10.1111/j.1467-8306.1994.tb01882.x

[jgrf21488-bib-0070] Townend, I. H. , Wang, Z. B. , & Rees, J. G. (2007). Millennial to annual volume changes in the Humber Estuary. Proceedings of the Royal Society A: Mathematical, Physical & Engineering Sciences, 463, 837–854. 10.1098/rspa.2006.1798

[jgrf21488-bib-0071] Umitsu, M. , Buman, M. , Kawase, K. , & Woodroffe, C. D. (2001). Holocene palaeoecology and formation of the Shoalhaven River deltaic‐estuarine plains, Southeast Australia. The Holocene, 11(4), 407–418. 10.1191/095968301678302841

[jgrf21488-bib-0072] Van de Lageweg, W. I. , Braat, L. , Parsons, D. R. , & Kleinhans, M. G. (2018). Controls on mud distribution and architecture along the fluvial‐to‐marine transition. Geology, 46(11), 971–974. 10.1130/g45504.1

[jgrf21488-bib-0073] Van der Spek, A. J. F. (1995). Reconstruction of tidal inlet and channel dimensions in the Frisian Middelzee, a former tidal basin in the Dutch Wadden Sea. Tidal Signatures in Modern and Ancient Sediments, 24, 239–258. 10.1002/9781444304138.ch16

[jgrf21488-bib-0074] Van der Spek, A. J. F. (1997). Tidal asymmetry and long‐term evolution of Holocene tidal basins in The Netherlands: Simulation of palaeo‐tides in the Schelde estuary. Marine Geology, 141(1–4), 71–90. 10.1016/s0025-3227(97)00064-9

[jgrf21488-bib-0075] Van der Wal, D. , Herman, P. M. J. , Forster, R. M. , Ysebaert, T. , Rossi, F. , Knaeps, E. , et al. (2008). Distribution and dynamics of intertidal macrobenthos predicted from remote sensing: Response to microphytobenthos and environment. Marine Ecology Progress Series, 367, 57–72. 10.3354/meps07535

[jgrf21488-bib-0076] Van der Wegen, M. , & Roelvink, J. A. (2008). Long‐term morphodynamic evolution of a tidal embayment using a two‐dimensional, process‐based model. Journal of Geophysical Research, 113(C3), 1–23. 10.1029/2006jc003983

[jgrf21488-bib-0077] Van Dijk, W. M. , Van de Lageweg, W. I. , & Kleinhans, M. G. (2013). Formation of a cohesive floodplain in a dynamic experimental meandering river. Earth Surface Processes and Landforms, 38(13), 1550–1565. 10.1002/esp.3400

[jgrf21488-bib-0078] Van Maren, D. S. , Oost, A. P. , Wang, Z. B. , & Vos, P. C. (2016). The effect of land reclamations and sediment extraction on the suspended sediment concentration in the Ems Estuary. Marine Geology, 376, 147–157. 10.1016/j.margeo.2016.03.007

[jgrf21488-bib-0079] Van Rijn, L. C. (1997). Sediment transport and budget of the central coastal zone of Holland. Coastal Engineering, 32(1), 61–90. 10.1016/s0378-3839(97)00021-5

[jgrf21488-bib-0080] Vos, P. (2015). Origin of the Dutch coastal landscape: Long‐term landscape evolution of The Netherlands during the Holocene, described and visualized in national, regional and local palaeogeographical map series. Barkhuis.

[jgrf21488-bib-0081] Vos, P. , De Koning, J. , & Van Eerden, R. (2015). Landscape history of the Oer‐IJ tidal system, Noord‐Holland (The Netherlands). Netherlands Journal of Geosciences, 94(4), 295–332. 10.1017/njg.2015.27

[jgrf21488-bib-0082] Wang, Z. B. , & Townend, I. H. (2012). Influence of the nodal tide on the morphological response of estuaries. Marine Geology, 291, 73–82. 10.1016/j.margeo.2011.11.007

[jgrf21488-bib-0083] Weisscher, S. A. H. , Boechat Albernaz, M. , Leuven, J. R. F. W. , Van Dijk, W. M. , Shimizu, Y. , & Kleinhans, M. G. (2020). Complementing scale experiments of rivers and estuaries with numerically modelled hydrodynamics. Earth Surface Dynamics, 8(4), 955–972. 10.5194/esurf-8-955-2020

[jgrf21488-bib-0084] Weisscher, S. A. H. , Shimizu, Y. , & Kleinhans, M. G. (2019). Upstream perturbation and floodplain formation effects on chute cutoff‐dominated meandering river pattern and dynamics. Earth Surface Processes and Landforms, 44(11), 2156–2169. 10.1002/esp.4638 31598027 PMC6774324

[jgrf21488-bib-0085] Woodroffe, C. D. , Rogers, K. , McKee, K. L. , Lovelock, C. E. , Mendelssohn, I. , & Saintilan, N. (2016). Mangrove sedimentation and response to relative sea‐level rise. Annual Review of Marine Science, 8, 243–266. 10.1146/annurev-marine-122414-034025 26407146

[jgrf21488-bib-0086] Zecchin, M. , Brancolini, G. , Tosi, L. , Rizzetto, F. , Caffau, M. , & Baradello, L. (2009). Anatomy of the Holocene succession of the southern Venice lagoon revealed by very high‐resolution seismic data. Continental Shelf Research, 29(10), 1343–1359. 10.1016/j.csr.2009.03.006

[jgrf21488-bib-0087] Zhou, Z. , Coco, G. , Jiménez, M. , Olabarrieta, M. , Van der Wegen, M. , & Townend, I. (2014). Morphodynamics of river‐influenced back‐barrier tidal basins: The role of landscape and hydrodynamic settings. Water Resources Research, 50(12), 9514–9535. 10.1002/2014wr015891

[jgrf21488-bib-0088] Zhou, Z. , Coco, G. , Townend, I. , Olabarrieta, M. , Van Der Wegen, M. , Gong, Z. , et al. (2017). Is “morphodynamic equilibrium” an oxymoron? Earth‐Science Reviews, 165, 257–267. 10.1016/j.earscirev.2016.12.002

