# Supplementary material for: Building and Raising Land: Mud and Vegetation Effects in Infilling Estuaries
Source: J Geophys Res Earth Surf. 2022 Jan 17;127(1):e2021JF006298. doi: 10.1029/2021JF006298 (PMC9286589; doi:10.1029/2021JF006298)
Supplement: Supplementary file 1 — Supporting Information S1 [file JGRF-127-0-s003.pdf]

Supplementary material to:
Building and raising land: mud and vegetation effects in infilling estuaries

Steven A.H. Weisscher*[1], Kim Van den Hoven[1], Harm-Jan Pierik[1], and Maarten G. Kleinhans[1]

[1]Faculty of Geosciences, Utrecht University, Princetonlaan 8A, 3584 CB, Utrecht, The Netherlands

December 20, 2021

Supplement 1: Validation of Nays2D flow modelling
Supplement 2: Pilot experiments in the mini-Metronome
Supplement 3: Algae and fungi protocol
Supplement 4: Cross-sections over the bay-head delta

*Corresponding author
Email address: s.a.h.weisscher@uu.nl
Tel. 0031 30 253 7641

Supplement 1: Validation of Nays2D flow modelling

This study did an extra validation of the hydrodynamic numerical model Nays2D for estuary scale experiments, in addition to the earlier validation by Weisscher et al. (2020). The reason for an extra validation was that the experimental setup of this study involved considerably larger tidal volumes than the estuary for which Nays2D was validated (Leuven et al., 2018; Weisscher et al., 2020). To this end, one Particle Imaging Velocimetry (PIV) measurement (cf. Leuven et al., 2018) was conducted in this study at the end of the experiment with only sand to create residual flow velocity maps over a tidal cycle. In addition, water blueness values were extracted from the overhead imagery, which were used as indicator of water depths over a tidal cycle.

The tidal signal along the estuary was well reproduced by the numerical model. This is evident from the tidal zonation maps in Figure 1, which are derivatives of water depth variations; supra-tidal areas remain dry over a tidal cycle, intertidal areas are only submerged during flood and subtidal areas are flooded the entire tidal cycle. The numerical model resulted in slightly less intertidal area in the downstream half of the estuary, for example at the intertidal bar at $x = 16$ m and at the seaward side of the main mid-channel bar at $x = 14$ m (Figure 1). In contrast, slightly more intertidal area was modelled for the upstream half of the estuary, for example at the small mid-channel bar at $x = 7.5$ m and for the bayhead delta $x < 4$ m. The largest differences between the two maps were due to shortcomings of the overhead cameras. These are the striping at $x[8-11]$ m that results in less intertidal area in the measured maps, and the lighting difference of the downstream-most camera that results in the sharp edge between tidal zonation at $x = 19$ m.

Modelled residual flow shows a largely similar pattern to the measured values: the model shows there are two distinct ebb-dominated channels in the downstream half of the estuary at either side of the basin. The lower channel shows a slightly meandering ebb-tidal channel with small pockets of flood-dominant flow, which correspond with flood barbs in the morphology.

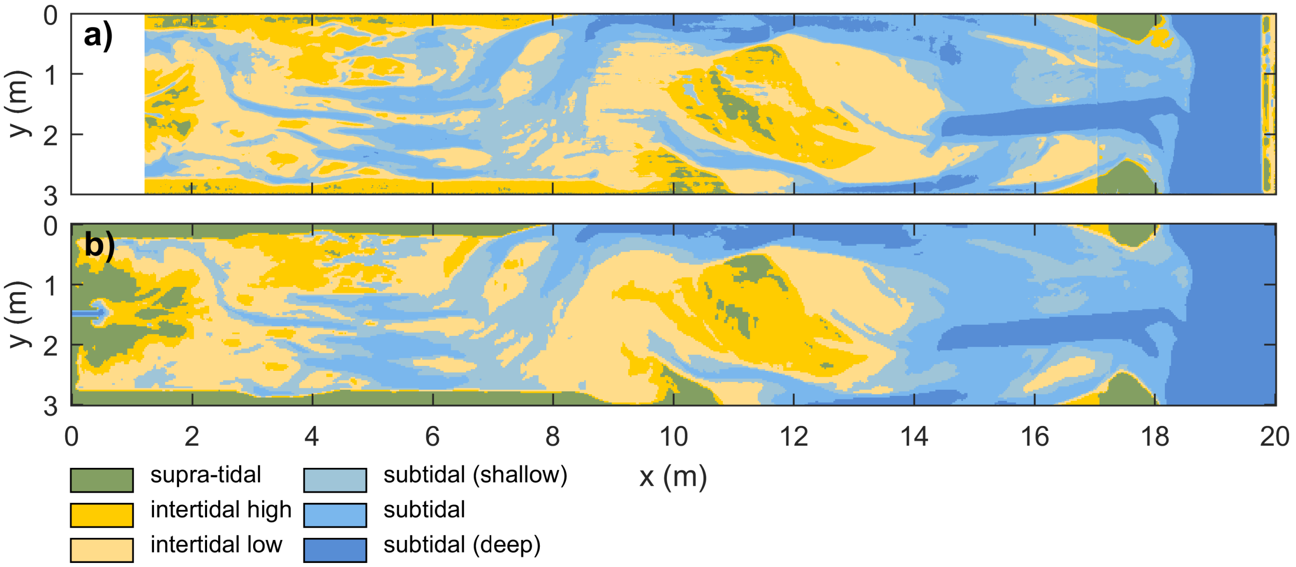

Figure 1: Tidal zonation maps of the end of the experiment with only sand based on (a) water colour saturation registered by overhead cameras and (b) numerically modelled flow in Nays2D. The upstream-most part of (a) was obstructed by the bridge and thus has no data. River inflow is at the left and the sea boundary is at the right.

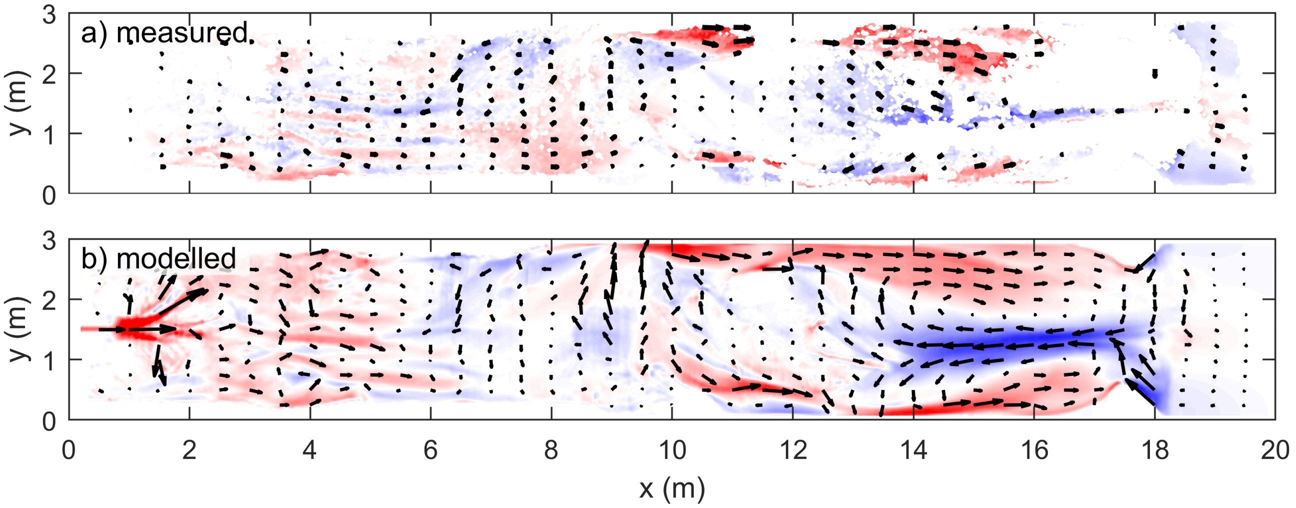

Figure 2: Residual tidal flow at the end of the experiment with only sand based on (a) Particle Imaging Velocimetry measurements and (b) modelled flow in the numerical model Nays2D. Residual flow measurements are only shown if more than three quarters of all measurements over a tidal cycle are available.

The flood-dominant tidal channel from the ebb-tidal delta into the estuary splits upon reaching the large mid-channel bar at $x = 14$ m. This is fairly similar with the measurements, although many in-situ measurements are missing. There are notable differences in the upstream half of the estuary. Firstly, there is a large region of slight ebb-dominance at $x = 8$ m, which is less distinct or absent in the modelled data. Additionally, the many parallel channels downstream of the bayhead delta ($x[2 - 6]$ m) are mostly ebb-dominant in the numerical model, whilst measurements indicate that about half of them are weakly flood-dominant.

Supplement 2: Pilot experiments in the mini-Metronome

Introduction

Preliminary to the Metronome experiments described in the main article, pilot runs were conducted in a smaller flume the mini-metronome. In the mini-metronome, a Holocene bar-built estuary was created to study the effect of vegetation on the formation of channels and bars in an evolving estuary. Different vegetation densities were studied to find the optimal vegetation input for the main Metronome experiments.

Methods

Three pilot runs were conducted in the flume the mini-metronome measuring 3.5 m by 1.2 m (developed by Kleinhans et al., 2014; 2015b; Fig. 3). Tilting the flume over the short central axis simulated a symmetrical tidal flow with a period of 34.0 s and an amplitude of 12.1 mm. A constant water level was maintained by continued in and outflow at the seaward boundary. At the river boundary, water enriched with sand was provided continuously. Once every tidal cycle, fluvial input was enriched with crushed walnut shells, representing silt and clay. Sand characteristics in the pilot runs were similar to characteristics in the Metronome, with median grain size d50 = 0.55 mm. However, walnut consisted of only two equal fractions: 0.20 mm and 1.2-1.7 mm. Sediment input per tidal cycle was 1.2 ml sand and 5.7 ml walnut.

In each simulation, initial morphology was a drowned river valley with two barriers next to the tidal inlet. The two barriers were locked in place by metal frames; hence the tidal inlet width was constant throughout the experiment. To represent the natural development of centuries to millennia each experiment was planned to run for 3,000 tidal cycles (Kleinhans et al., 2015a). The control experiment lacked vegetation. The other two experiments had vegetation introduced into an evolving estuary. Seeds of two species, *Medicago sativa* and *Lotus pedunculatus*, entered the basin hydrochorously at the river inlet every 500 tidal cycles. The two vegetation experiments differed in the number of seeds introduced at the river: 25.0 g *Medicago* seeds and 25.0 g *Lotus* seeds per vegetation cycle that resulted in a sparse cover and 80.0 g *Medicago* seeds and 80.0 g *Lotus* seeds per vegetation cycle that resulted in a dense cover. Due to a very high vegetation density, the dense vegetation experiment was aborted after 1,800 tidal cycles.

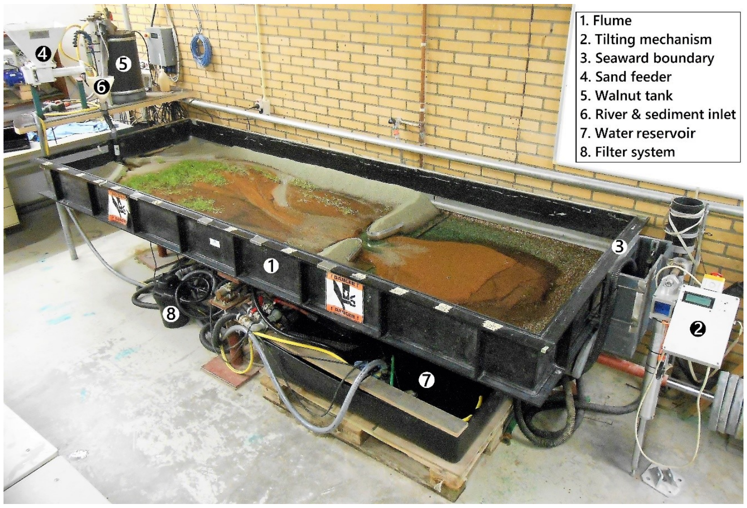

Figure 3: Setup of the flume the mini-metronome (after MSc thesis by K. van den Hoven).

Vegetation growth and morphological development was studied quantitatively and qualitatively. Time-lapse photography in phase with the tide captured the entire flume in four positions every 4th tidal cycle. Photographs represented flood, ebb, high water and low water. In addition, the estuary was carefully drained to scan the bed elevation and obtain detailed

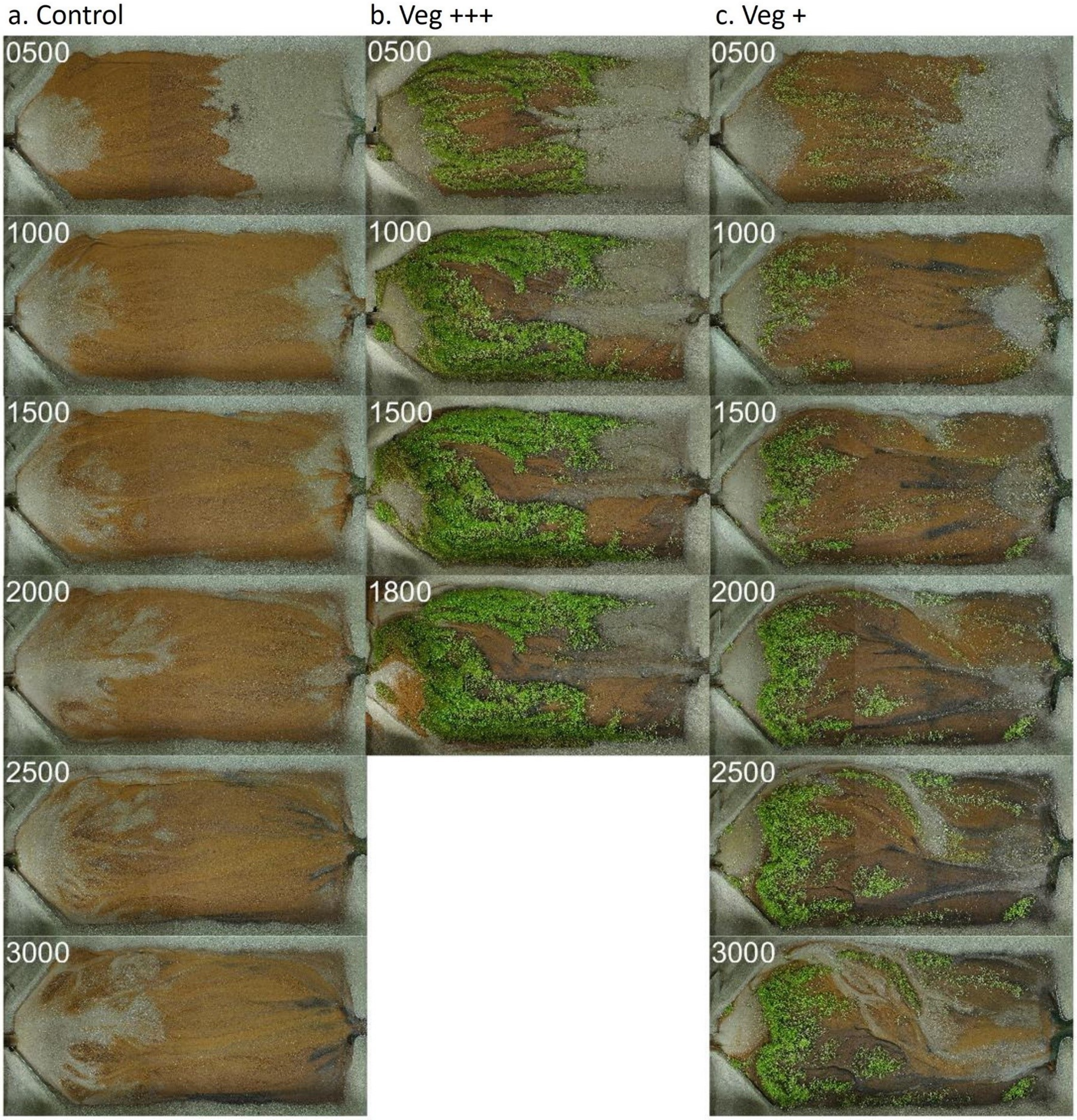

Figure 4: Basin top view with water drained from the flume in (a) the control run, (b) dense vegetation run, and (c) sparse vegetation run. In each photograph, left = river inflow, right = barriers with tidal inlet, and top left corner depicts the cumulative number of tidal cycles that the experiment was run, with the end stage in the final photograph of each column. Top row photographs were taken just before tide and river flow was started again after the first seeding (after MSc thesis by K. van den Hoven).

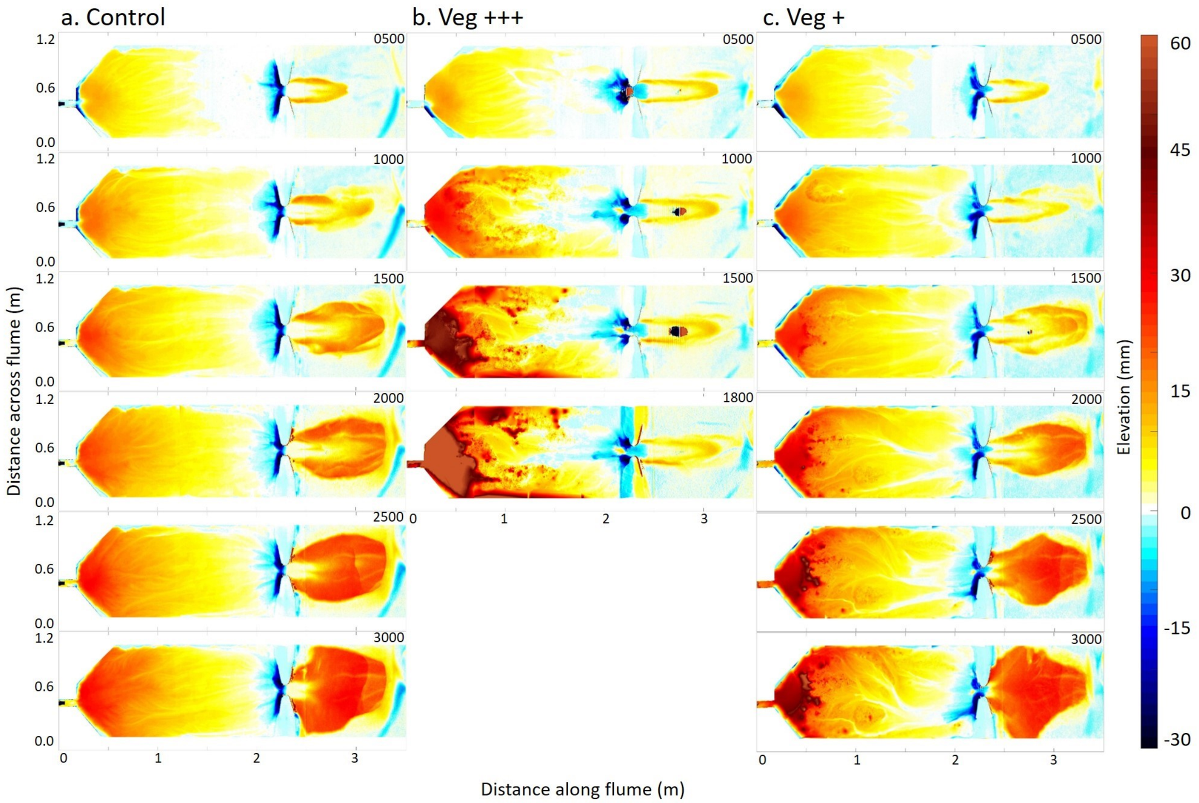

Figure 5: Bed elevation change for (a) the control run, (b) dense vegetation, and (c) sparse vegetation. Blue = erosion, white = unchanged, and yellow-brown = deposition. The darkest patches in (b) and (c) are due to incomplete correction for vegetation. In each figure, left = river inflow, right = the sea, and top right corner depicts the cumulative number of tidal cycles that the experiment was run, with the end stage in the final figure of each column. A small part of the flume was not captured by the zSnapperTM, so elevation data for the uttermost left and right side of the flume (top and bottom in each figure) is missing (after MSc thesis by K. van den Hoven).

photographs, after time = 250 and after time = 500 tidal cycles, and subsequently at intervals of 100 tidal cycles. Bed elevation was scanned with a ViALUX 3d scanner consisting of a fringe projector and calibrated camera system, together termed: zSnapperTM. Photographs were taken with a digital single-lens reflex camera on a fixed frame above the flume.

Results

Initial morphological development was similar in all three pilot runs. During the first 500 tidal cycles walnut was deposited up to halfway the basin, small channels run parallel from the river inflow, and a sandy bay-head delta and ebb tidal delta evolved (top row in Figs. 4, 5). The introduction of vegetation changed the estuarine morphology (Figs. 4-6). Overall, vegetation establishment led to an increase in channel width and depth, and a decrease in braiding index. Sediment deposition per time step was highest during the dense vegetation run. The very dense vegetation captured sediment already in proximity of the river inflow. This is visualised by the highly elevated bay-head delta and a small ebb tidal delta (Figs. 5, 6). With sparse vegetation, sediment could be transported throughout the tidal basin, so channels and bars increased in size and a large ebb tidal delta evolved (Figs. 5, 6). After 2,500 tidal cycles, the main fluvial channel connected to the tidal channel.

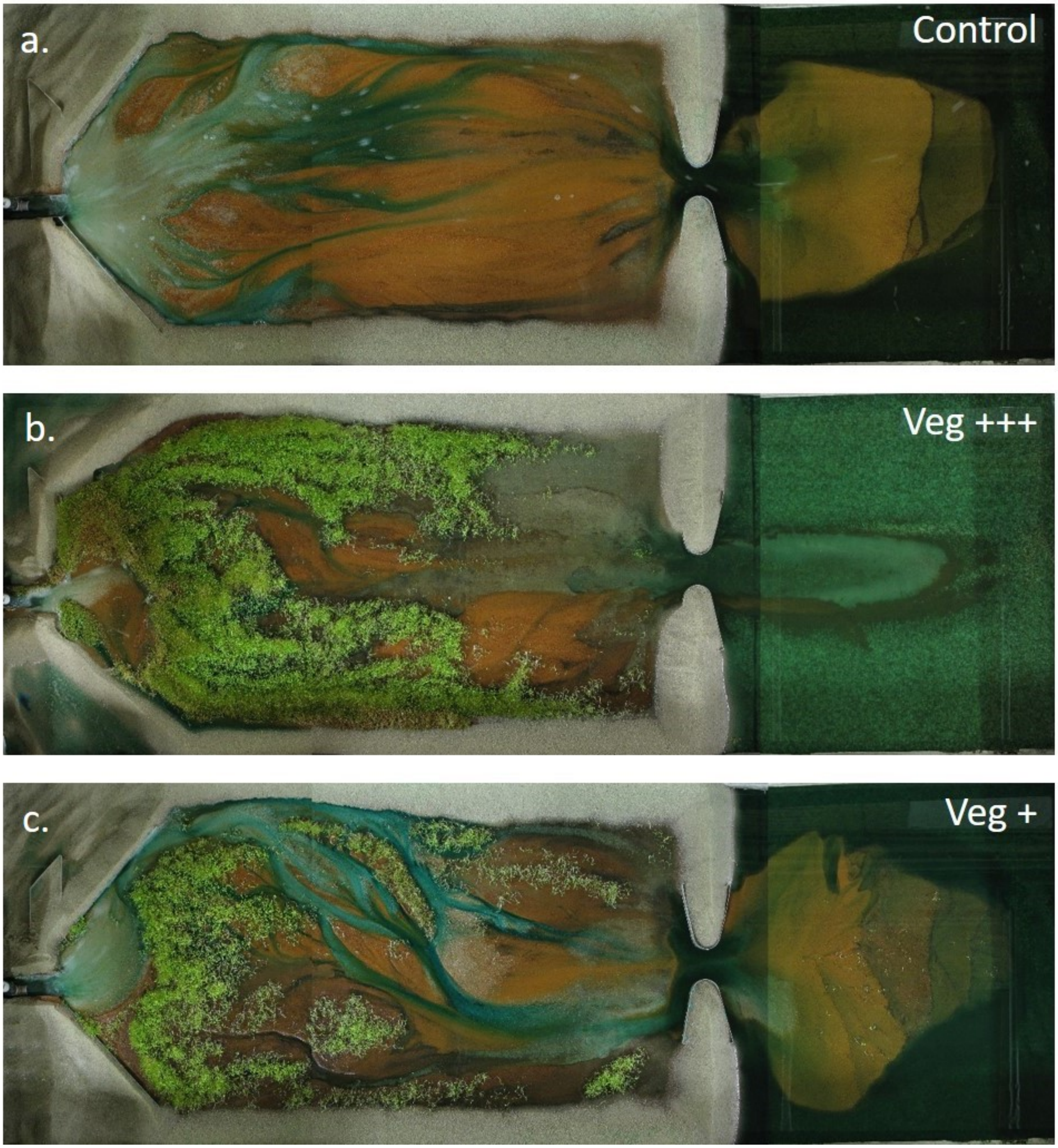

Figure 6: Flume top view for the (a) control run at 2,810 tidal cycles, (b) dense vegetation run at 1,595 tidal cycles, and (c) sparse vegetation run at 2,968 tidal cycles. Left is river inflow, right is sea with ebb tidal delta. Blue dyed water indicates channels (after MSc thesis by K. van den Hoven).

Towards designing the Metronome experiments

The pilot runs in the mini-metronome flume provided insight into the eco-engineering effects of vegetation in Holocene estuary development. In the presence of vegetation, the estuarine system narrowed down when compared to the basin without vegetation. Vegetation captured sediment, so downstream sediment export reduced. Locally, vegetation patches created shelter for sediment deposition while areas with dense vegetation patches were static and hardly evolved over

time. Vegetation density influenced estuary development. On the one hand, dense vegetation (Veg +++) led to a static system in which downstream sediment transport was inhibited. On the other hand, sparse vegetation (Veg +) created a dynamic system in which channels and bars evolved and adapted. The estuary with sparse vegetation evolved in a river-dominated and tide-dominated part (Fig. 7).

The pilot runs contributed to the experimental set-up in the Metronome. In the pilot runs, differentiation of vegetation settlement was limited as both types of seeds were able to settle in similar locations, so in the Metronome experiments the smaller *Veronica beccabunga* was added to represent pioneer vegetation settlement. The pilot dense vegetation run showed it is important not to provide too much vegetation into the evolving estuary at once. Therefore, relatively small amounts of seeds were sowed through the river and tidal inlet in the Metronome. In addition, discharge was increased to enable continuous river flow through dense vegetation. In contrast to the pilot runs, extreme bayhead delta formation was limited in the Metronome by reducing fluvial sediment supply and adding marine sediment supply. Marine sediment was supplied by erosion of the sand barriers and by walnut input at the tidal inlet.

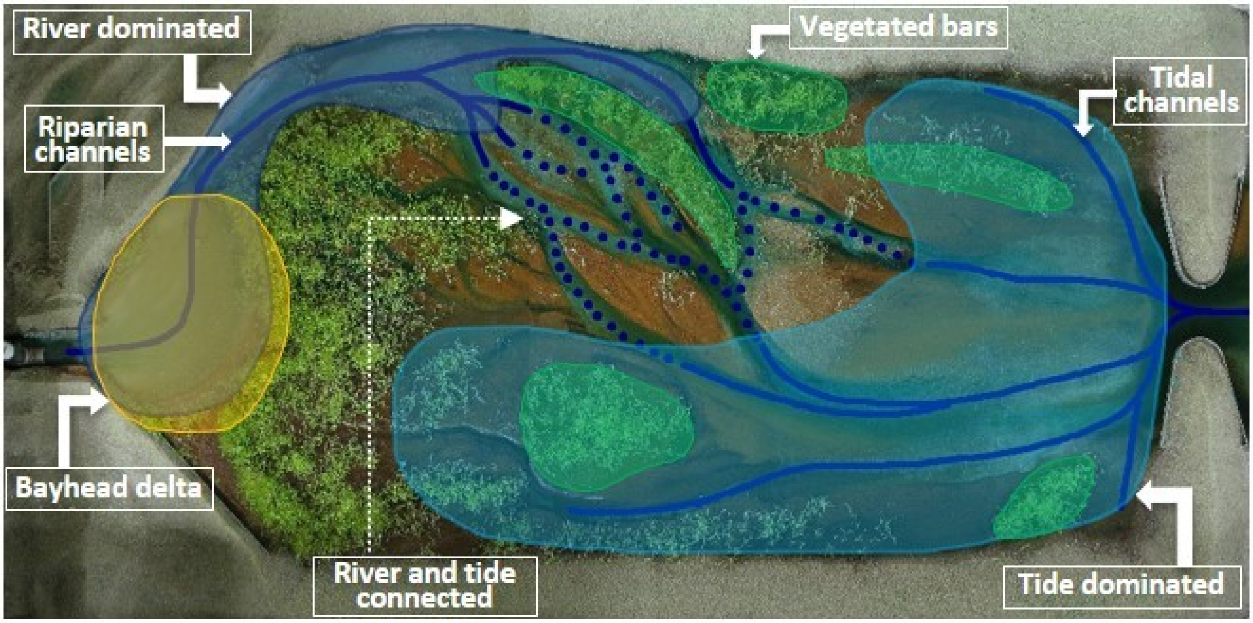

Figure 7: Estuary overview of experiment with sparse vegetation at tidal cycle 2,968 (after MSc thesis by K. van den Hoven).

Supplement 3: Algae and fungi protocol

Algal blooms were suppressed in the Metronome. 100 ml of anti-algae Algofin was added to the flume twice a week. Also, a bio-filter with active carbon was installed and outflow filters at either end of the flume were cleaned every time the flume was drained for scans so as to mitigate the growth of algae. Overall, algae had an insignificant effect on the morphodynamics, in line with Friedkin bank erosion tests under similar conditions in previous studies Braat et al. (2019).

In contrast, fungal growth posed a more serious problem to the morphological development in experiments with crushed nutshell. Fungi flourished in shallow regions of the estuary, especially when the flume was not tilting during scans and during days of vegetation sprouting. Consequently, the tops of tidal bars became very cohesive, more than could be attributed to the nutshell alone. This complication resulted in large scarps up to 2 cm when part of these bars were eroded, which occurred predominantly, but not exclusively, on tidal bars covered by nutshell in the upstream part of the estuary. Additionally, the threshold for motion was enhanced in the upstream part of the estuary by the fungi as the experiment progressed, which likely contributed to lower dynamics in later stages of development.

The growth of fungi was counteracted in two ways. Firstly, Pimafix™ was added to the nutshell feeders to prevent fungi from flourishing inside the crushed nutshell. Second, the estuary bed was sprayed with water with low concentrations of tea tree oil and Pimafix™. Such spraying was done three times in the second experiment (sand and mud); intervals of no flow were relatively short, so fungal growth did not take large proportions. However, in the third experiment (sand, mud and vegetation) tilting was off repeatedly for four days to allow vegetation sprouting. To prevent fungi growth during the no-tilting periods, the upstream part of the estuary and brownish spots were sprayed every time the flume had been drained.

Supplement 4: Cross-sections over the bay-head delta

Figures 8 and 9 show cross-sections over the bay-head delta for x = 80, 120 and 160 cm. The mud experiment (Figure 8) clearly shows alternating layers of mud and sand. Mud abundance increases towards the valley edges and in the downstream direction on the bay-head delta. This trend is in contrast to the mud and vegetation experiment (Figure 9), where vegetation diverted flow and caused large spatial differences in mud and sand deposition. Therefore, there are fewer clear continuous mud layers in the subsurface and more local thick mud deposits, for example on the left in all cross-sections in Figure 9.

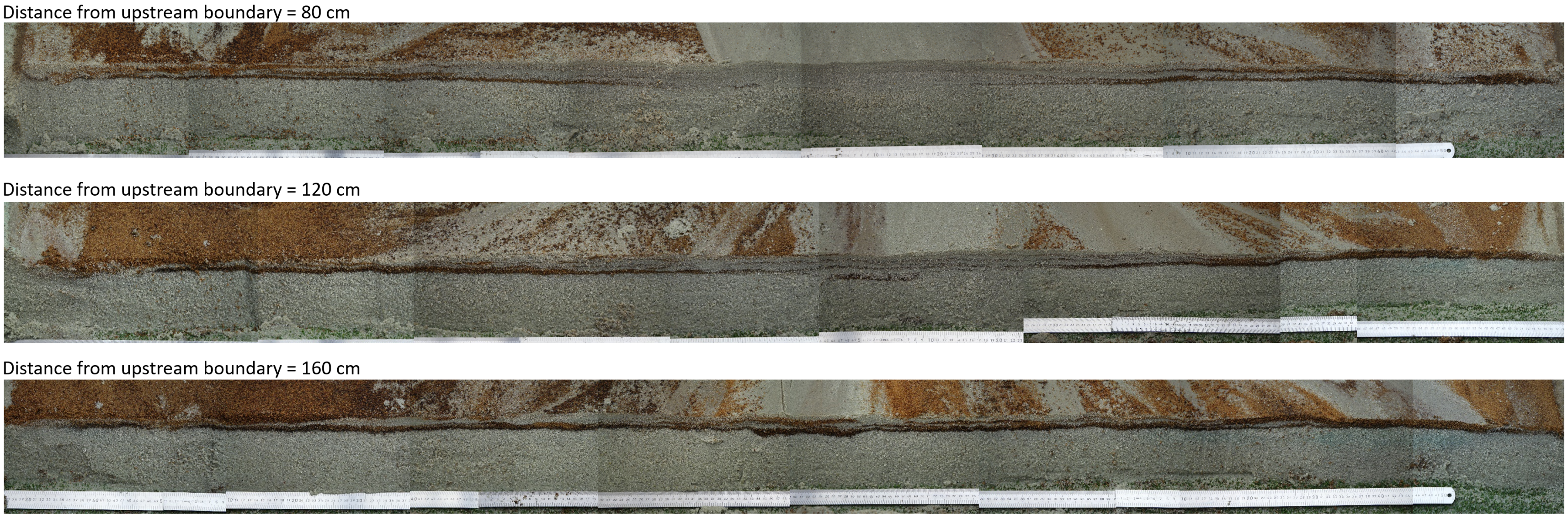

Figure 8: Cross-sections over the bay-head delta at x = 80, 120 and 160 cm in the mud experiment. Photos were taken facing the upstream direction. The top part of the cross-sections is the pristine surface.

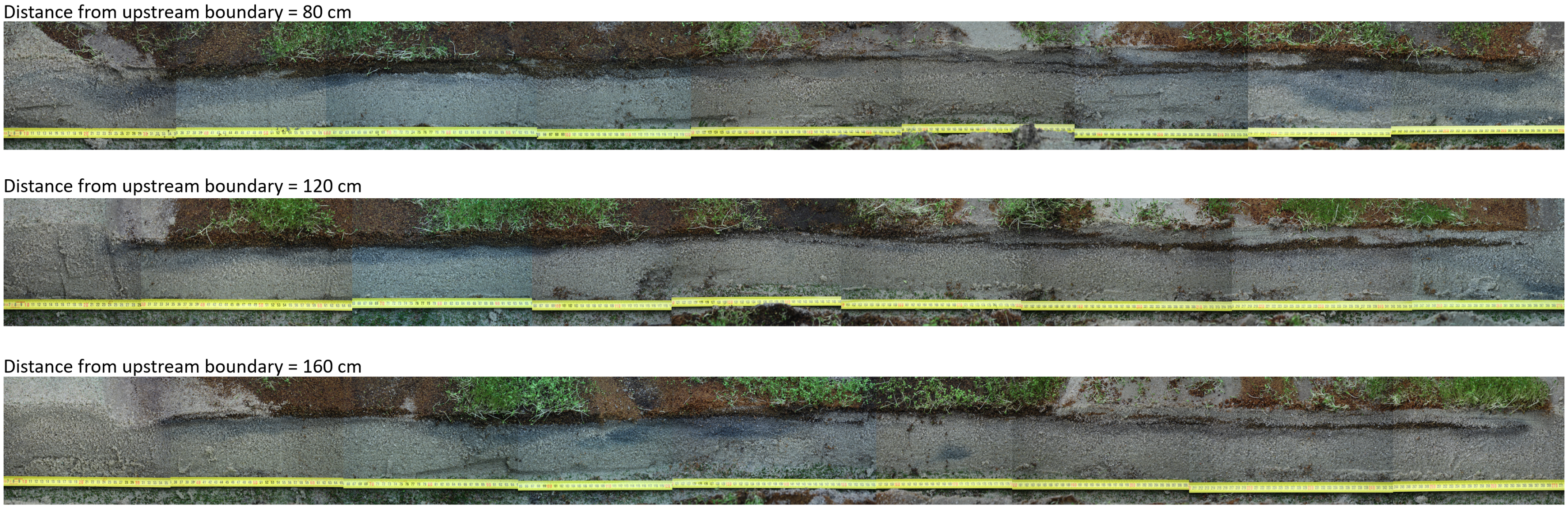

Figure 9: Cross-sections over the bay-head delta at x = 80, 120 and 160 cm in the mud and vegetation experiment. Photos were taken facing the upstream direction. The top part of the cross-sections is the pristine surface.

References

Braat L, Leuven JRFW, Lokhorst IR, Kleinhans MG. 2019. Effects of estuarine mudflat formation on tidal prism and large-scale morphology in experiments. *Earth Surface Processes and Landforms*, 44(2):417–432.

Kleinhans MG, Van Rosmalen TM, Roosendaal C, Van der Vegt M. 2014. Turning the tide: mutually evasive ebb-and flood-dominant channels and bars in an experimental estuary. *Advances in Geosciences*, 39:21–26.

Kleinhans MG, Braudrick C, Van Dijk WM, Van de Lageweg WI, Teske R, Van Oorschot M. 2015a. Swiftness of biomorphodynamics in Lilliput- to Giant-sized rivers and deltas. *Geomorphology*, 244:56–73. doi: 10.1016/j.geomorph.2015.04.022.

Kleinhans MG, Van Scheltinga RT, Van Der Vegt M, Markies H. 2015b. Turning the tide: Growth and dynamics of a tidal basin and inlet in experiments. *Journal of Geophysical Research: Earth Surface*, 120(1):95–119.

Leuven JRFW, Braat L, Van Dijk WM, De Haas T, Van Onselen EP, Ruessink BG, Kleinhans MG. 2018. Growing forced bars determine nonideal estuary planform. *Journal of Geophysical Research: Earth Surface*, 123(11):2971–2992.

Weisscher SAH, Boechat Albernaz M, Leuven JRFW, Van Dijk WM, Shimizu Y, Kleinhans MG. 2020. Complementing scale experiments of rivers and estuaries with numerically modelled hydrodyanmics. *Earth Surface Dynamics*, 8(4):955–972.
